# Molecular and Environmental Elucidation of Heavy Metal Transfer in *Tilia* spp.: From Soil Systems to Herbal Infusions Across Urban–Forest Gradients

**DOI:** 10.3390/ijms27041856

**Published:** 2026-02-14

**Authors:** Petrică Tudor Moțiu, Călin Gheorghe Pășcuț, Szilárd Bartha, Camelia Elena Moga, Octavian Berchez, Ioana Andra Vlad, Ioan Tăut, Florin Alexandru Rebrean, Florin-Dumitru Bora

**Affiliations:** 1 Department of Forestry and Forest Engineering, University of Oradea, 1 University Street, 410087 Oradea, Romania; tudormotiu@gmail.com (P.T.M.); pascutcalin@yahoo.com (C.G.P.); barthaszilard10@yahoo.com (S.B.); cameliamoga90@gmail.com (C.E.M.); 2 Department of Agriculture and Horticulture, University of Oradea, 1 University Street, 410087 Oradea, Romania; berchez_octavian@yahoo.com; 3 Department of Food Engineering, University of Oradea, 1 University Street, 410087 Oradea, Romania; iana_andravlad@yahoo.co.uk; 4 Department of Forestry, Faculty of Forestry and Cadastre, University of Agricultural Sciences and Veterinary Medicine, 3-5 Mănăștur St., 400372 Cluj-Napoca, Romania; ioan.taut@usamvcluj.ro; 5 ”Marin Drăcea” National Institute for Research and Development in Forestry Cluj, 65 Horea Street, 400372 Cluj-Napoca, Romania; 6 Viticulture and Oenology Department, Advanced Horticultural Research Institute of Transylvania, Faculty of Horticulture and Business in Rural Development, University of Agricultural Sciences and Veterinary Medicine Cluj-Napoca, 3-5 Mănăștur Street, 400372 Cluj-Napoca, Romania; 7 Laboratory of Chromatography, Advanced Horticultural Research Institute of Transylvania, Faculty of Horticulture and Business for Rural Development, University of Agricultural Sciences and Veterinary Medicine, 400372 Cluj-Napoca, Romania

**Keywords:** *Tilia* spp., heavy metal translocation, herbal infusions, molecular–environmental biomonitoring, urban–forest gradients, estimated daily intake (EDI), toxicological risk assessment, environmental exposure, multi-matrix analysis

## Abstract

Understanding the pathways through which heavy metals accumulate in medicinal plants and enter herbal infusions is essential for linking environmental quality with human exposure. This study investigated multi-matrix metal transfer in *Tilia* spp. along an urban–forest gradient by quantifying twelve elements (Pb, Cd, Zn, Cu, Ni, Cr, Mn, Co, As, Hg, Al, and V) in soil, bark, leaves, flowers, and corresponding infusions using inductively coupled plasma mass spectrometry and by estimating daily intake for different age groups based on EFSA default body weights and two consumption scenarios (150 and 400 mL day^−1^). The results revealed clear spatial patterns, with significantly higher metal loads in urban sites and a consistent transfer from environmental compartments to plant tissues and infusions. Mn, Al, Pb, and Cd exhibited the highest extractability, leading to elevated estimated daily intakes in young children, identified as the most vulnerable group due to their lower body mass. However, all exposure values remained below EFSA and JECFA toxicological reference limits, while As and Hg were undetectable in all infusions. These findings indicate that *Tilia* infusions contribute minimally to overall dietary metal exposure and confirm *Tilia* spp. as reliable bioindicators of soil- and airborne metal deposition, supporting the safe consumption of linden tea under realistic intake conditions.

## 1. Introduction

Medicinal and aromatic plants used for the preparation of herbal infusions represent an important component of human nutrition and traditional phytotherapeutic practices worldwide [1,2,3]. As shown by the comprehensive synthesis of Alawadhi et al. (2024) [4], these species are capable of accumulating heavy metals in different vegetative organs as a result of complex interactions with environmental factors [5,6]. Metal contamination arises from both natural sources, such as soil geochemical background, and anthropogenic inputs, including traffic-related atmospheric deposition, industrial emissions, agricultural practices, and post-harvest handling [4,5,6]. Therefore, medicinal plants function not only as therapeutic resources but also as sensitive indicators of environmental metal burdens relevant for biomonitoring and human exposure assessment.

The presence of heavy metals in plant materials intended for human consumption raises important food safety concerns, particularly under conditions of regular or long-term intake [4]. As highlighted in the global analysis reported in [4], metals such as lead, cadmium, nickel, and aluminum may exert cumulative toxic effects, with chronic exposure being associated with neurological, cardiovascular, renal, and metabolic disorders [7,8,9,10]. In herbal infusions, these risks may be further enhanced by hot water extraction processes that mobilize bioavailable metal fractions from plant tissues, increasing oral exposure [4]. Therefore, herbal infusions represent a key interface between environmental contamination and human health, warranting integrated toxicological and molecular–environmental investigation.

Urban environments are commonly characterized by elevated burdens of heavy metals, particularly lead (Pb) and cadmium (Cd), resulting from sustained anthropogenic activities such as vehicular traffic and industrial emissions [11,12]. These elements accumulate in urban soils, persist over long periods, and are readily transferred to biotic compartments [13,14]. Due to their high toxicity and bioaccumulation potential, chronic exposure to Pb and Cd has been associated with neurological, cardiovascular, and renal impairments [15,16,17]. Consequently, their presence in urban vegetation requires particular attention when plant-derived materials are intended for human consumption, including medicinal and aromatic applications [4,18].

Species of the genus *Tilia* are widely used in urban monitoring frameworks due to their stable physiological performance under diverse environmental conditions and their widespread distribution in European cities [18,19]. Their large foliar surface and high capacity to retain airborne particulate matter make *Tilia* species particularly suitable for assessing spatial variability in urban environments [20,21]. Several studies have shown that heavy metal accumulation in *Tilia* foliage is predominantly controlled by atmospheric wet and dry deposition rather than root uptake, rendering leaves effective indicators of urban air quality and potential human exposure pathways [20,21,22,23,24]. Consequently, *Tilia* spp. have been extensively employed as biomonitors of atmospheric trace metal pollution in urban and industrial settings, providing reproducible information on spatial and temporal contamination patterns [11,22,25,26,27].

To quantify potential human exposure to heavy metals through the consumption of plant-based infusions, daily dietary intake assessment is a key component of health risk evaluation. Standardized metrics, including estimated daily intake (EDI), non-carcinogenic hazard quotient (HQ), and carcinogenic risk (CR), are widely applied in food safety and environmental health studies [4,28]. These indices combine measured metal concentrations in plant-derived products with physiological and exposure parameters, enabling the assessment of health risks across different population groups [4,28,29,30,31,32].

Children represent a particularly vulnerable group with respect to dietary exposure to heavy metals. Due to their lower body mass, the consumption of identical volumes of herbal infusions results in higher metal intake on a body-weight-adjusted basis compared to adults. Therefore, the explicit inclusion of pediatric populations in exposure assessment scenarios is essential for ensuring a conservative and health-protective risk characterization [33,34,35,36].

The present study aims to characterize heavy metal dynamics in *Tilia* species along an urban–forest gradient and to elucidate their multi-matrix transfer from soil and bark to leaves, flowers, and ultimately herbal infusions consumed by humans. Although *Tilia* spp. are widely used as urban biomonitors, integrated studies linking soil and atmospheric metal sources with their transfer into consumable infusions and age-specific dietary exposure remain limited. By quantifying elemental concentrations across environmental and plant compartments and calculating the Estimated Daily Intake (EDI) for different age groups, this study evaluates the contribution of linden infusions to dietary metal exposure and their toxicological relevance, while also assessing the suitability of *Tilia* species as bioindicators of urban metal pollution.

## 2. Results

### 2.1. Physicochemical Variability of Soils Beneath Tilia spp. Across Urban–Forest Habitats

**Figure 1:**
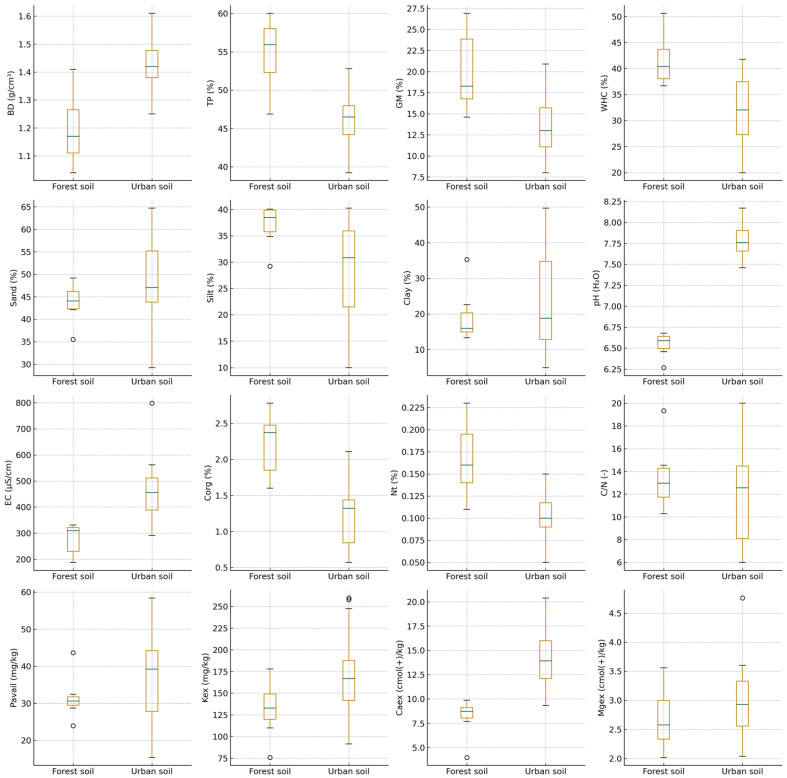
Comparative distribution of physicochemical soil properties beneath *Tilia* spp. across urban and forest habitats.

The physicochemical properties of the 33 soil samples exhibited pronounced variability across sites supporting *Tilia tomentosa*, *Tilia cordata*, and *Tilia platyphyllos*. Despite their spatial proximity, soils associated with individual species displayed distinct structural and chemical characteristics. Soils beneath *T. tomentosa*, which accounted for the majority of samples, showed the widest range of physical variability. Bulk density ranged from 1.04 g/cm^3^ (TL-S01) at a forest-associated site to 1.61 g/cm^3^ (TL-S07) in an intensively urbanized location, with corresponding total porosity values spanning 60.00% (TL-S01) to 39.24% (TL-S07). Water-related parameters also varied markedly within this species, with gravimetric moisture between 8.02% (TL-S27) and 25.99% (TL-S08), and water-holding capacity ranging from 20.00% (TL-S29) to 53.48% (TL-S08). Textural composition under *T. tomentosa* was similarly heterogeneous, from sand-dominated substrates such as TL-S16 (64.71% sand) to clay-enriched soils like TL-S25 (49.67% clay). Overall, soils associated with Tilia species differed consistently between forest and urban habitats, with clear contrasts in bulk density, porosity, moisture-related parameters, and texture (Figure 1). Although marker size is constant and does not encode measured values, we acknowledge that the graphical layout may visually suggest size-related differences. Therefore, all figures were revised to ensure strictly uniform marker dimensions and to eliminate any potential interpretation of marker area as a quantitative variable. Concentrations are represented exclusively by the color scale and axis values.

Soils supporting *T. cordata* displayed comparatively narrower physical ranges while still preserving clear contrasts across sampling habitats. Bulk density varied between 1.21 g/cm^3^ (TL-S09) and 1.48 g/cm^3^ (TL-S13/S14), whereas total porosity ranged from 44% to 54%. Moisture-related indicators were moderately elevated in forest-associated *T. cordata* soils, with water-holding capacity reaching 39–44% (TL-S01, TL-S09), and lower values recorded in urban soils such as TL-S11 and TL-S13. Soil texture remained predominantly loamy, with sand fractions between 37% and 54% and clay contents between 18% and 44%.

In contrast, soils beneath *T. platyphyllos* (TL-S08, TL-S12, TL-S21, TL-S31, TL-S32) exhibited more uniform physical characteristics. Bulk density ranged from 1.28 to 1.48 g/cm^3^, while total porosity varied between 44% and 52%. Water-holding capacity spanned from 29.46% (TL-S32) to 53.48% (TL-S08), with TL-S08 standing out as the most moisture-retentive *T. platyphyllos* site. Textural patterns were generally balanced, with moderate proportions of sand (45–55%), silt (28–40%), and clay (9–22%). Detailed physical and chemical properties of individual soil samples are provided in Supplementary Table S1a.

**Figure 2:**
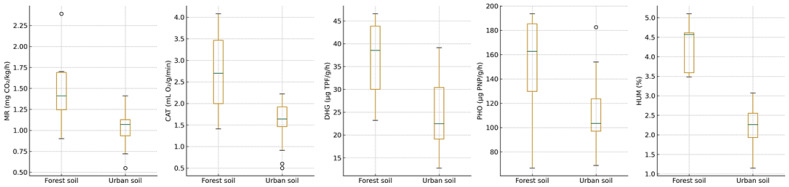
Comparative distribution of soil biological activity indicators beneath *Tilia* spp. across urban and forest habitats.

Chemical parameters also differed among species-associated soils. Under *T. tomentosa*, pH(H_2_O) ranged from 6.27 (TL-S09) to 8.17 (TL-S14), spanning the full regional gradient from slightly acidic forest soils to alkaline urban substrates. Electrical conductivity reached its maximum at TL-S14 (798.6 µS/cm), likewise beneath *T. tomentosa*. Soils supporting *T. cordata* exhibited pH values predominantly between 6.66 and 7.73 and moderate EC levels (220–559 µS/cm), whereas *T. platyphyllos* soils maintained consistently alkaline conditions (pH 7.06–8.12) with EC ranging from 202 to 442 µS/cm. Soil biological activity parameters further displayed clear contrasts between forest and urban habitats across the sampled sites (Figure 2). Site-specific values of soil biological and biochemical indicators are summarized in Supplementary Table S1b.

Organic carbon exhibited the highest concentrations in soils beneath *T. tomentosa*, reaching a maximum of 2.78% (TL-S01), whereas urban *T. tomentosa* soils showed the lowest values, ranging between 0.57% and 0.80% (TL-S27, TL-S10). Soils associated with *T. cordata* ranged from 0.71% (TL-S13) to 2.45% (TL-S22), while *T. platyphyllos* displayed intermediate organic carbon levels (0.77–1.47%). Total nitrogen followed similar trends (0.05–0.23%), and the C/N ratio remained within 6–20 across all species. Nutrient availability varied markedly, particularly under *T. tomentosa*, where plant-available phosphorus reached 58.43 mg/kg (TL-S10) and exchangeable potassium peaked at 197.72 mg/kg (TL-S02). The highest exchangeable calcium concentration was recorded under *T. platyphyllos* (259.88 cmol(+)/kg, TL-S31), whereas forest-associated *T. cordata* soils exhibited the lowest values (7.69 cmol(+)/kg, TL-S09). Exchangeable magnesium ranged from 1.43 cmol(+)/kg (TL-S18) under *T. tomentosa* to 4.76 cmol(+)/kg (TL-S12) under *T. platyphyllos*.

**Figure 3:**
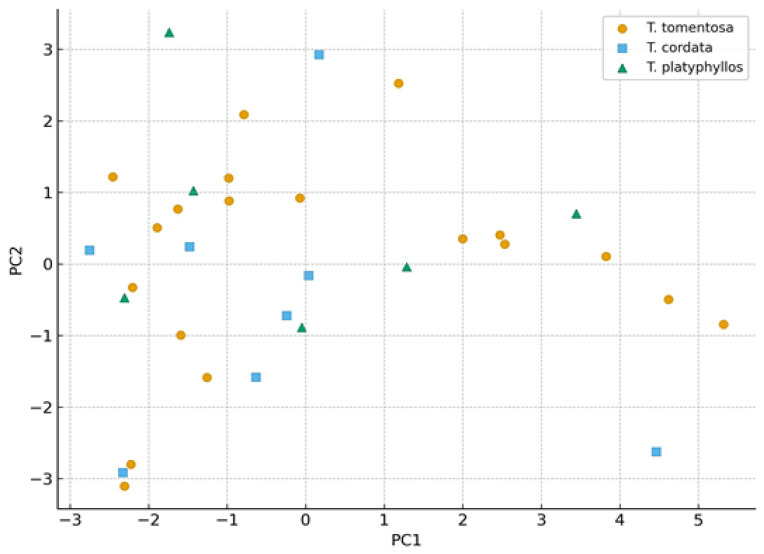
Multivariate separation of *Tilia* species according to soil physicochemical properties revealed by PCA.

Principal component analysis (PCA) revealed distinct multivariate patterns in soil properties across the sampled sites. The score plot indicates a partial separation of soil samples according to *Tilia* species along the first two principal components (Figure 3). Samples associated with *T. tomentosa* exhibited the widest dispersion, reflecting greater variability in soil physicochemical and biological conditions, whereas samples linked to *T. cordata* and *T. platyphyllos* tended to form more compact clusters. The relative positioning of samples along PC1 and PC2 thus reflects differences in combined soil characteristics rather than the influence of individual parameters, highlighting both species- and habitat-related gradients in soil conditions across the study area.

### 2.2. Distribution of Heavy Metals in Soils Beneath Tilia spp.

**Figure 4:**
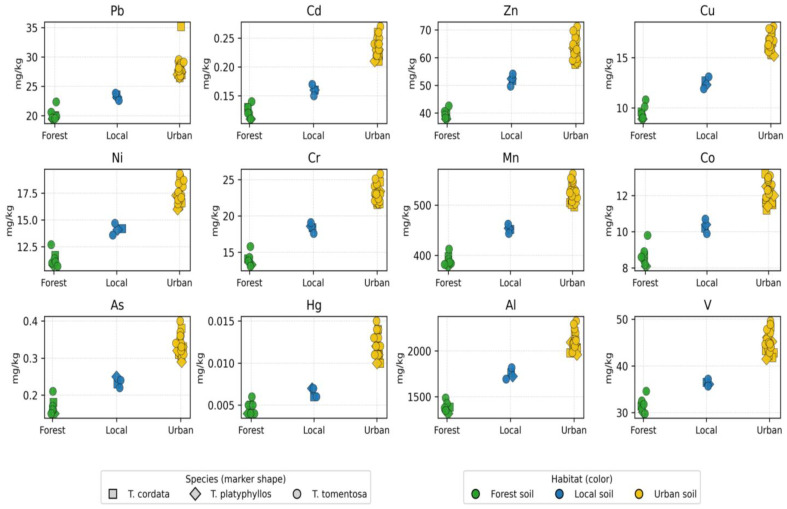
Habitat- and species-related variation in soil heavy metal concentrations beneath *Tilia* species in the Oradea region.

Table S2 provides a comprehensive dataset of priority heavy metals quantified in soil samples collected beneath three *Tilia* species (*T. tomentosa*, *T. cordata*, and *T. platyphyllos*) in the Oradea region. A total of 33 soil samples were analyzed, encompassing three habitat types—urban, forest, and local soils—and capturing representative variability in land use influence and atmospheric deposition patterns. The descriptive statistics and spatial distribution of the analyzed elements reveal substantial heterogeneity dependent on both environmental context and tree-species association. These patterns are visually summarized in Figure 4, which illustrates multi-element variation across habitats and species.

Lead (Pb) concentrations exhibited the greatest variability among the monitored toxic metals, ranging from approximately 19.5 to 35.2 mg/kg (mean ≈ 25.9 mg/kg). The moderate relative standard deviation (RSD ≈ 14–15%) indicates marked spatial heterogeneity, largely associated with anthropogenic influence. The highest Pb levels were consistently observed in urban soils, suggesting cumulative deposition related to traffic emissions, industrial combustion residues, and legacy contamination. In contrast, forest soils reflected near-background geochemical levels (≈20 mg/kg), indicating minimal anthropogenic interference.

Cadmium (Cd), although present only at trace levels (mean ≈ 0.20 mg/kg, range 0.11–0.27 mg/kg), exhibited relatively high variability, supporting the inference that Cd originates from localized sources such as tire wear particles, fertilizers, and municipal aerosol inputs. Similarly, arsenic (As) and mercury (Hg) occurred at low but detectable concentrations (≈0.15–0.40 mg/kg for As and ≈0.004–0.015 mg/kg for Hg), with high coefficients of variation (CV > 25%) indicating sensitivity to micro-scale atmospheric deposition processes. Their presence, despite low absolute abundance, underscores the environmental relevance of monitoring persistent pollutants even at sub-mg levels, given their recognized bioaccumulation potential.

In contrast, essential nutrient elements and lithogenic constituents—including Zn, Cu, Ni, Cr, Mn, Al, and V—were present at substantially higher concentrations, reflecting a dominant geogenic control and the baseline mineralogical composition of the soils. Zinc (Zn) ranged between 38 and 71 mg/kg (mean ≈ 57 mg/kg), representing the most abundant of the micronutrients and indicating contributions from both soil parent material and surface enrichment in urban locations. Copper (Cu) varied from 9 to 18 mg/kg and exhibited a clear urban elevation trend, consistent with particulate emissions from traffic-related mechanical wear and industrial aerosols.

Nickel (Ni) and chromium (Cr) exhibited intermediate concentrations (≈11–19 mg/kg and ≈13–26 mg/kg, respectively), suggesting a combined influence of natural geological sources and gradual anthropogenic accumulation. Manganese (Mn) and aluminum (Al) recorded the highest abundances among the analyzed elements, reaching approximately 490–560 mg/kg for Mn and exceeding 2000 mg/kg for Al in urban sites, thereby confirming their dominant geochemical role and their suitability as baseline indicators of the soil mineral matrix. Vanadium (V) displayed a comparable spatial pattern, with higher concentrations in urban soils, likely associated with fuel–oil combustion residues and industrial particulate deposition.

When the data were stratified by species, *T. tomentosa* exhibited the highest accumulation levels for most of the analyzed elements (Pb, Zn, Cu, Ni, Mn, and Al), whereas *T. platyphyllos* generally showed the lowest concentrations, indicating species-specific efficiency in element retention and differential biomonitoring potential. Variations in metal distribution across habitats (urban versus forest) and among species further highlight the strong influence of land use type and biological traits on soil metal enrichment, thereby reinforcing the suitability of *Tilia* species as sensitive ecological indicators for urban environmental quality monitoring.

A habitat-based comparison revealed a consistent concentration gradient across most analytes, following the order Urban > Local > Forest soils. Urban soils exhibited the highest mean concentrations (e.g., Pb = 27.68 mg/kg, Zn = 62.41 mg/kg, Mn = 516.09 mg/kg), confirming pronounced urban pressure and enhanced deposition of particulate-bound metals. Forest soils represented the least contaminated matrix (e.g., Pb = 20.02 mg/kg, Zn = 40.87 mg/kg), serving as a reliable proxy for the regional geochemical background. Local soils displayed transitional patterns, likely reflecting partial urbanization and moderate anthropogenic influence.

Differences among *Tilia* species were less pronounced than habitat-driven variation but remained scientifically meaningful. Soils beneath *T. tomentosa* exhibited slightly higher concentrations of Pb, Zn, and Mn compared to those associated with *T. cordata* and *T. platyphyllos*, while *T. platyphyllos* generally displayed intermediate values across most elements. These distinctions may be attributed to species-specific differences in root system architecture, canopy morphology influencing dry deposition interception, and spatial distribution patterns within the landscape matrix.

### 2.3. Heavy Metal Accumulation in the Bark of Tilia spp.

Table S3 provides a detailed geochemical dataset describing the concentrations of priority heavy metals retained in bark samples collected from three *Tilia* species (*T. tomentosa*, *T. cordata*, and *T. platyphyllos*) across the Oradea metropolitan region. A total of 34 bark samples were analyzed from three contrasting habitat categories—urban, forest, and local sites—capturing a representative gradient of anthropogenic pressure and atmospheric deposition intensity.

**Figure 5:**
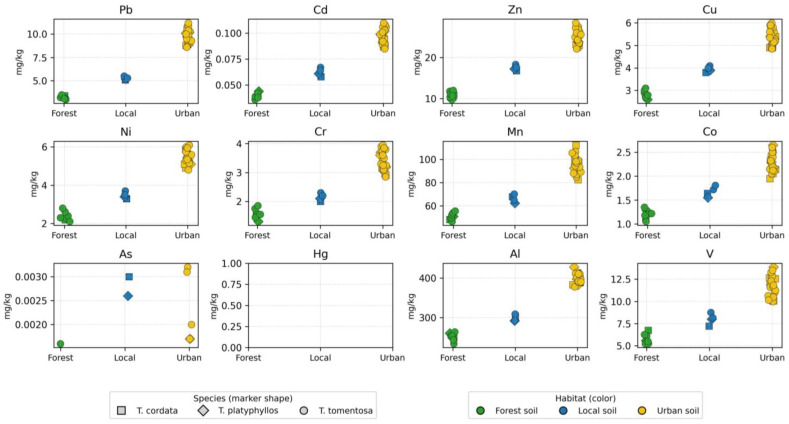
Habitat- and species-related variation in bark heavy metal concentrations of *Tilia* species in the Oradea region.

The descriptive statistics reveal pronounced elemental variability associated with differences in environmental context, emission exposure, and species-specific bark morphology and sorption characteristics. This heterogeneity underscores the diagnostic potential of bark as a passive biomonitoring matrix for particulate-bound metal pollution and is graphically illustrated in Figure 5, which depicts multi-element variation across habitats and species. Mercury (Hg) concentrations were below the analytical detection limit (BLD) in all flower samples; therefore, no quantifiable values are displayed in the Hg panel.

Lead (Pb) exhibited the greatest variability among the toxic heavy metals retained in bark, with concentrations ranging from 3.0 to 11.2 mg/kg (mean ≈ 7.87 mg/kg; RSD ≈ 36%). The consistently elevated Pb levels recorded in urban environments indicate a strong influence from vehicle exhaust particulates, brake and tire abrasion products, and industrial aerosol emissions. In contrast, the markedly lower Pb concentrations observed in forest zones (≈3.25 mg/kg) closely reflect regional atmospheric background conditions, reinforcing the suitability of forest sites as reference benchmarks. The pronounced spatial gradient in Pb further supports its role as a sensitive tracer of transportation-related atmospheric contamination.

Cadmium (Cd) occurred at much lower absolute concentrations (mean ≈ 0.081 mg/kg, range 0.036–0.110 mg/kg) yet exhibited substantial relative variability (RSD ≈ 30%), indicating a strong dependence on localized emission microenvironments. This pattern is consistent with Cd inputs originating from automobile component wear, municipal waste incineration, and fertilizer-derived particulate entrainment. Arsenic (As) and mercury (Hg) were detected at extremely low levels, typically marginally above or below analytical detection limits. Nevertheless, despite their minimal concentrations, the observed spatial variability suggests episodic deposition of persistent contaminants with long atmospheric residence times and potential for chronic bioaccumulation.

The highest concentrations among all analyzed elements corresponded to lithogenic and nutrient constituents such as Mn, Al, Zn, and Cu, reflecting both the geochemical background and enhanced particulate deposition in urban environments. Zinc concentrations ranged from 9.8 to 28.4 mg/kg (mean ≈ 21.1 mg/kg), indicating combined contributions from physiologically mediated accumulation and pronounced sensitivity to motorized traffic emissions. Copper (Cu), varying between 2.6 and 6.0 mg/kg, likewise exhibited a clear urban enrichment pattern consistent with metallic abrasion products, industrial aerosols, and mechanical wear processes. Manganese (Mn) and aluminum (Al) displayed the highest overall abundances (Mn mean ≈ 83.2 mg/kg; Al ≈ 357.7 mg/kg), demonstrating that bark serves as an efficient collector of mineral particulate matter entrained in the air column.

Spatial analysis confirmed a well-defined hierarchical gradient in metal accumulation—Urban > Local > Forest—with urban bark displaying significantly elevated mean concentrations (e.g., Pb = 9.72 mg/kg, Mn = 94.01 mg/kg, Al = 401.52 mg/kg), indicative of intensified atmospheric deposition processes in densely trafficked areas. Local environments exhibited intermediate profiles reflecting partial anthropogenic exposure, whereas forest habitats showed the lowest concentrations, functioning as reliable baselines for regional atmospheric conditions.

Species-related differences, although less pronounced than habitat-driven contrasts, revealed functionally relevant patterns. *T. tomentosa* exhibited the highest accumulation capacity for most metals (Pb, Zn, Cu, Mn, and Al), whereas *T. platyphyllos* generally showed the lowest metal retention efficiency, with *T. cordata* occupying intermediate positions. These differences can be attributed to interspecific variation in bark porosity, surface roughness, chemical composition, and canopy structure, which collectively regulate the interception and binding of airborne particulates.

### 2.4. Heavy Metal Accumulation in Leaves of Tilia spp.

**Figure 6:**
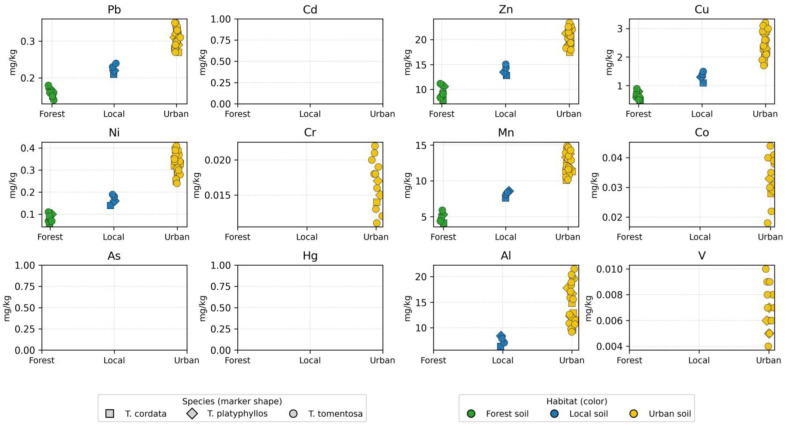
Habitat- and species-related variation in leaf heavy metal concentrations of *Tilia* species in the Oradea region.

Table S4 provides a detailed dataset describing the concentrations of selected priority heavy metals retained in leaf samples collected from three *Tilia* species (*T. tomentosa*, *T. cordata*, and *T. platyphyllos*) across the Oradea region. A total of 34 leaf samples were analyzed from contrasting habitat categories—urban, forest, and local sites—capturing a representative gradient of atmospheric exposure and anthropogenic pressure. The descriptive statistics (mean, SD, RSD, min–max) reveal moderate to high variability for most elements, reflecting differences in atmospheric deposition intensity, local emission sources, and species-specific foliar sorption characteristics. This heterogeneity underscores the diagnostic capacity of *Tilia* leaves as a sensitive biomonitoring matrix for particulate-bound metal pollution and is visually summarized in Figure 6, which illustrates multi-element variation as a function of habitat type and species.

Lead (Pb) exhibited pronounced spatial variability in leaf tissues, with concentrations ranging from 0.14 to 0.35 mg/kg (mean ≈ 0.27 mg/kg; RSD ≈ 25%). Elevated Pb levels were characteristic of urban habitats (mean ≈ 0.31 mg/kg), clearly contrasting with those measured in forest leaves (≈0.16 mg/kg). This pattern reflects enhanced deposition and surface retention of Pb-bearing particulates in traffic-influenced environments, likely originating from vehicular exhaust residues, brake and tire wear, and urban resuspension dust. Conversely, the lower Pb concentrations in forest samples approximate background atmospheric levels, confirming the suitability of forest stands as reference sites in regional air quality assessments and supporting the use of foliar Pb as a tracer of traffic-related pollution.

Zinc (Zn) and copper (Cu), which serve dual roles as essential micronutrients and atmospheric contaminants, exhibited the highest concentrations among the analyzed trace metals in leaf tissue. Zinc ranged from 7.9 to 23.4 mg/kg (mean ≈ 17.36 mg/kg), while Cu varied between 0.5 and 3.2 mg/kg (mean ≈ 1.97 mg/kg). Both elements showed pronounced enrichment in urban sites (Zn ≈ 19.82 mg/kg, Cu ≈ 2.38 mg/kg) compared with forest habitats (Zn ≈ 9.60 mg/kg, Cu ≈ 0.66 mg/kg), reflecting combined contributions from physiological uptake and deposition of traffic- and industry-derived aerosols.

For Cd, As, and Hg, all measurements were below the analytical detection limit (BLD); therefore, no data points are shown in the corresponding panels (Figure 6).

Nickel (Ni) exhibited a similar spatial pattern, with urban leaves accumulating nearly fourfold higher concentrations (≈0.31 mg/kg) than forest leaves (≈0.08 mg/kg), pointing to mixed contributions from fuel combustion products and metallurgical or industrial activities.

In contrast, cadmium (Cd), arsenic (As), mercury (Hg), and, in many cases, cobalt (Co) were predominantly below the limit of detection (LOD) in the analyzed leaf samples. Their generally negligible concentrations indicate that, under the studied conditions, atmospheric inputs of these highly toxic elements were relatively low compared with major micronutrients and lithogenic metals. Nevertheless, the occasional detection of Co in urban leaves, together with the measurable accumulation of Mn and Al, underscores the capacity of foliage to integrate signals from both local and regional air-mass histories, particularly for elements associated with fine particulate matter.

Manganese (Mn) and aluminum (Al) exhibited some of the highest abundances among the investigated elements (Mn mean ≈ 10.21 mg/kg; Al ≈ 10.82 mg/kg), with pronounced spatial contrasts. In urban habitats, mean Mn concentrations (~12.13 mg/kg) were approximately threefold higher than those in forest leaves (~3.99 mg/kg), whereas Al was clearly enriched only in urban foliage (≈14.70 mg/kg), remaining effectively below detection in forest samples. These patterns indicate that Mn and Al accumulation in leaves is strongly influenced by the deposition of crustal material, construction dust, and road-derived mineral particulates, which are more abundant and more frequently resuspended in urban settings. Vanadium (V), although present at very low levels (mean ≈ 0.0029 mg/kg), was likewise more prevalent in urban leaves than in forest sites, consistent with its association with fuel–oil and diesel combustion.

When the data were stratified by habitat class, a clear hierarchical gradient in foliar metal accumulation emerged, following the order Urban > Local > Forest for most analytes. Urban leaf samples exhibited systematically elevated mean concentrations of Pb, Zn, Cu, Ni, Mn, Al, and V, indicating a strong influence of traffic emissions, industrial activities, and road dust resuspension. Local environments displayed intermediate signatures, consistent with mixed land use and moderate anthropogenic exposure, whereas forest habitats showed the lowest metal levels, effectively representing regional background conditions for airborne metal inputs.

Species-related differences, although less pronounced than habitat-driven contrasts, revealed functionally meaningful patterns in foliar metal retention. *T. tomentosa* generally exhibited the highest accumulation capacity for several elements (Zn, Cu, Mn, and Al), whereas *T. platyphyllos* showed slightly lower or comparable values, with *T. cordata* often occupying an intermediate position. These interspecific variations likely arise from differences in leaf surface microstructure, cuticle properties, trichome density, and canopy architecture, which collectively govern interception efficiency, retention time, and the potential resuspension of deposited particles.

### 2.5. Heavy Metal Accumulation in Tilia Flowers

**Figure 7:**
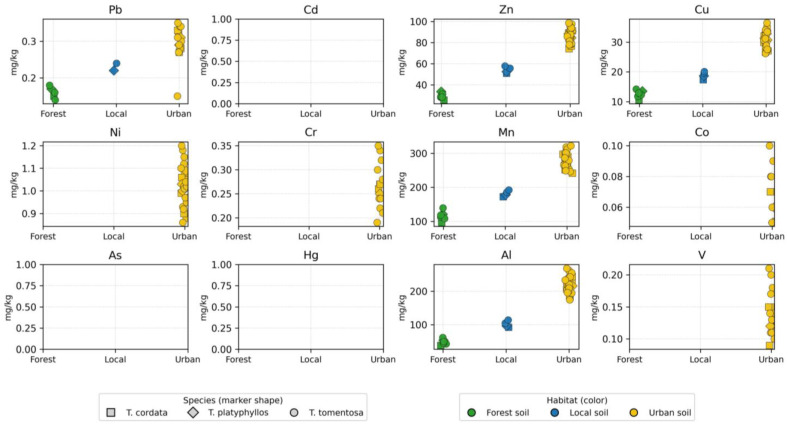
Habitat- and species-related variation in flower heavy metal concentrations of *Tilia* species in the Oradea region.

Heavy metal concentrations measured in *Tilia* flower samples from different habitats in the Oradea region are summarized in Table S5 and illustrated in Figure 7. For Cd, As, and Hg, all flower sample concentrations were below the analytical detection limit (BLD); consequently, no data points are displayed in the corresponding panels of Figure 7. Overall, Zn, Mn, and Al were the dominant elements, with concentration ranges of 25.9–98.6 mg/kg, 96.5–322.3 mg/kg, and 38.2–268.3 mg/kg, respectively. Copper (Cu) and nickel (Ni) occurred at intermediate levels, whereas Pb, Co, Cr, and V were detected at trace concentrations. Cadmium (Cd), arsenic (As), and mercury (Hg) were below the limit of detection (LOD) in most samples (Table S5). The predominance of Zn, Mn, and Al reflects both physiological accumulation processes and the influence of atmospheric deposition on floral tissues. In contrast, the consistently low levels of highly toxic elements such as Cd, As, and Hg suggest a limited transfer of these contaminants into reproductive plant organs under the studied environmental conditions.

Clear habitat-related differences were observed (Figure 7), with urban samples consistently exhibiting higher concentrations of most metals compared with forest sites. Mean Zn and Cu concentrations increased from approximately 30 mg/kg and 12 mg/kg in forest habitats to over 70 mg/kg and 30 mg/kg in urban areas, respectively. The most pronounced contrasts were recorded for Mn and Al, with maximum urban values exceeding 320 mg/kg and 260 mg/kg. Species-related differences were less marked than habitat effects: the distributions of *Tilia tomentosa*, *Tilia cordata*, and *Tilia platyphyllos* largely overlapped within the same habitat category (Figure 7), although *T. tomentosa* exhibited slightly higher maximum values for several elements in urban environments (Table S5). Trace elements such as Co, Cr, and V were detected primarily in urban samples and occurred sporadically across species, remaining absent or below the limit of detection (LOD) in forest sites. Overall, habitat type emerged as the primary factor controlling heavy metal concentrations in *Tilia* flowers.

### 2.6. Heavy Metal Mobilization into Tilia Flower Infusions Under Variable Brewing Conditions

**Figure 8:**
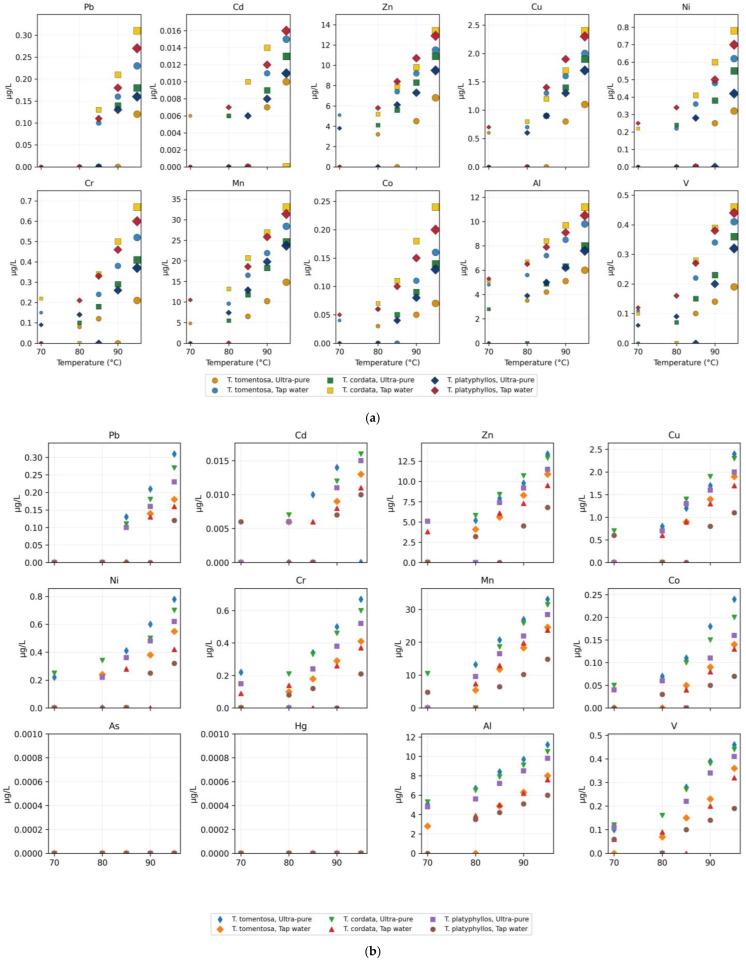
(**a**) Mobilization of heavy metals in *Tilia* flower infusions prepared with ultra-pure water under variable brewing temperatures; (**b**) Mobilization of heavy metals in *Tilia* flower infusions prepared with tap water under variable brewing temperatures.

In the applied experimental design, infusion time was not varied independently. Each brewing temperature was associated with a predefined infusion duration representative of common domestic preparation practices (70 °C/5 min and 95 °C/15 min). Consequently, temperature and time represent a combined brewing condition, as shown in Figure 8.

**Figure 9:**
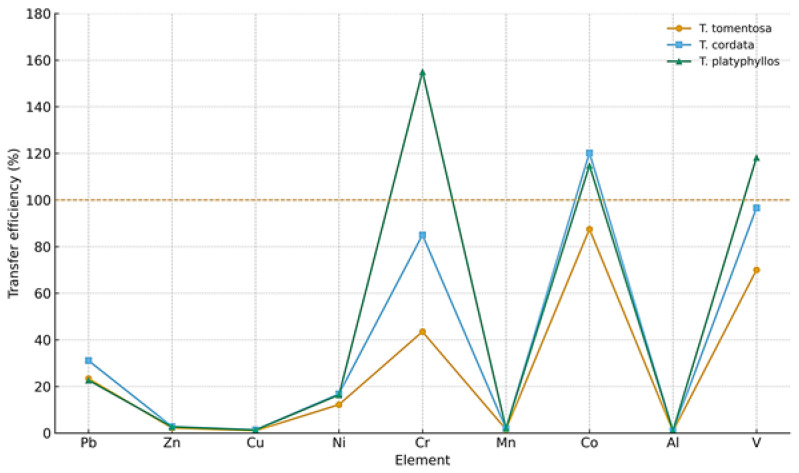
Comparative transfer efficiency of heavy metals from *Tilia tomentosa*, *Tilia cordata*, and *Tilia platyphyllos* into infusions. The dashed line represents the reference threshold (100%) used to evaluate transfer efficiency.

Table S6 and Figure 8 provide a detailed characterization of priority heavy metal mobilization into aqueous infusions prepared from *Tilia* flowers, highlighting the interactive effects of species identity, brewing temperature, infusion time, and water type on extraction efficiency and final concentration profiles. Direct comparison between the two standardized brewing scenarios (70 °C/5 min vs. 95 °C/15 min) indicates consistently higher elemental concentrations under the intensive extraction conditions. For most elements, concentrations increased from <LOD or low values at 70 °C/5 min to clearly quantifiable levels at 95 °C/15 min. When both values were quantifiable, the intensive brewing scenario generally resulted in approximately 2–5-fold higher concentrations (Table S6), confirming that metal mobilization into Tilia infusions is enhanced under higher temperatures and longer infusion times. The multi-element distribution visualized in Figure 9 reveals clear extraction trends across experimental treatments, confirming that metal solubility is primarily governed by the combined effect of brewing temperature–time conditions, followed by water mineralization and species-specific floral traits.

Among the toxic metals, Pb exhibited a pronounced temperature-dependent release pattern, with concentrations increasing progressively from below the limit of detection (LOD) at 70 °C to maximum values of approximately 0.31 µg/L at 95 °C (INF-C10; Table S6). The highest levels were associated with infusions prepared using tap water rather than ultrapure water, indicating a combined influence of enhanced desorption kinetics and ionic competition at elevated temperatures. Species-related differentiation was also evident, with peak values observed in *T. cordata* (0.31 µg/L; INF-C10), followed by *T. platyphyllos* (0.27 µg/L; INF-P10) and *T. tomentosa* (0.23 µg/L; INF-T10), reflecting differences in floral micromorphology and particulate adsorption capacity.

Mobilization of heavy metals into Tilia flower infusions as a function of brewing temperature, water type, and species. Infusion time was fixed for each temperature according to the standardized brewing protocol (70 °C/5 min and 95 °C/15 min) and therefore is not represented as an independent variable (Figure 8a).

Figure 8b illustrates systematic changes in heavy metal concentrations in *Tilia* flower infusions across predefined brewing scenarios that combine temperature and infusion time to reflect realistic domestic preparation practices rather than independent experimental variables. Across all elements, a consistent increase in concentrations was observed when moving from mild extraction conditions (70 °C/5 min) to intensive extraction conditions (95 °C/15 min), indicating enhanced metal mobilization under combined higher thermal input and prolonged contact time. Elements such as Mn, Zn, and Al showed the most pronounced responses to intensified brewing, reflecting their higher mobility within floral tissues and greater susceptibility to aqueous extraction under realistic household conditions. In contrast, potentially toxic trace metals such as Pb and Cd were generally present at low concentrations, frequently near or below detection limits under mild brewing, and became quantifiable mainly under intensive extraction conditions, while remaining below international drinking water guideline values.

Infusions prepared with tap water consistently exhibited higher elemental concentrations than those prepared with ultrapure water, highlighting the role of water chemistry and ionic strength in facilitating metal release. Additionally, species-specific differences were evident, with *Tilia cordata* generally displaying higher elemental transfer into infusions compared to *T. tomentosa* and *T. platyphyllos*, underscoring the influence of botanical characteristics on metal mobilization. Overall, the figure demonstrates that heavy metal release into *Tilia* infusions is governed by the combined temperature–time brewing scenario, water type, and species identity, supporting the interpretation of the results within a realistic consumer use framework rather than as isolated effects of individual preparation parameters.

These trends are consistent with the solid-phase elemental burdens reported in Table S5, where flowers collected from urban habitats exhibit higher Pb contents (up to 0.35 mg/kg) than those from forest environments, supporting atmospheric deposition as the dominant enrichment mechanism. Zinc (Zn) and copper (Cu) were the most readily extractable elements, in agreement with their high physiological mobility and elevated abundance in floral tissues. Zinc concentrations ranged from 3.2 to 13.4 µg/L, showing sharp increases with both temperature and infusion time, particularly in tap water extractions at 95 °C (e.g., INF-C10). Copper exhibited a comparable trend, increasing from below the limit of detection (LOD) to 2.4 µg/L (INF-C10), with release patterns closely mirroring Zn mobilization behavior. These results parallel the solid-phase levels reported in Table S5, where Zn reached 98.6 mg/kg and Cu 36.5 mg/kg in urban floral tissues, indicating strong transferability into infusion matrices.

Nickel (Ni) and chromium (Cr) displayed lower absolute mobility but followed the same thermally driven enhancement, peaking at 0.78 µg/L for Ni (INF-C10) and 0.67 µg/L for Cr (INF-C10). Their occurrence primarily in tap water extractions confirms the role of competitive ionic displacement and supports their anthropogenic association, consistent with elevated concentrations in urban floral tissues (Table S5: Ni up to 1.20 mg/kg; Cr up to 0.35 mg/kg). Manganese (Mn) demonstrated the greatest extraction variability, reaching 33.1 µg/L under high-temperature tap water infusions (INF-C10; Figure 9). This pronounced gradient correlates with the exceptionally high Mn content in dried flowers (Table S5 mean ≈ 239.43 mg/kg; up to 318.1 mg/kg in urban samples), confirming Mn as a dominant mobile fraction highly sensitive to environmental loading. Aluminum (Al) and vanadium (V) also displayed measurable extraction, rising to 11.2 µg/L and 0.46 µg/L, respectively, in tap water preparations at 95 °C (INF-C10), consistent with their known association with construction particulates and fossil-fuel combustion.

As demonstrated by Table S6 and Figure 8, metal mobilization into *Tilia* flower infusions is strongly governed by both environmental origin and extraction parameters, following a consistent spatial gradient in which urban samples exhibit the highest released concentrations, local samples show intermediate values, and forest samples the lowest levels (Urban > Local > Forest). Extraction responses also differed significantly among species, with *T. cordata* displaying the greatest release potential, followed by *T. platyphyllos* and *T. tomentosa* (*T. cordata* > *T. platyphyllos* > *T. tomentosa*), indicating that species-specific floral surface morphology and particulate retention efficiency influence elemental solubility. These findings demonstrate that brewing temperature, water mineralization, and the environmental history of floral tissues act synergistically to regulate metal transfer into consumable infusions, thereby determining the final chemical composition and variability of medicinal *Tilia* beverages.

### 2.7. Transfer Efficiency of Heavy Metals from Tilia Flowers to Infusions

Figure 9 does not resolve the influence of individual brewing parameters, but instead integrates the overall transfer efficiency of each element across the applied temperature–time conditions, allowing for comparison among *Tilia* species.

Herbal infusions were prepared by adding 1.00 g ± 0.01 g of dried *Tilia* flowers to 250 mL of water, and the transfer efficiency of each metal was calculated based on the concentrations measured in the aqueous extracts. The results (Figure 9) revealed clear element-dependent extraction patterns across the three *Tilia* species.

Overall, Zn, Cu, and Mn exhibited the lowest transfer efficiencies, remaining below 10% in all species. Lead (Pb) also showed limited extraction, with values ranging between 20 and 30%. In contrast, substantially higher transfer efficiencies were observed for Ni, Cr, Co, Al, and V, with several elements reaching values above 80% and, in some cases, exceeding 100% in *T. cordata* and *T. platyphyllos*. The three species displayed consistent trends: *T. platyphyllos* registered the highest transfer efficiencies for Cr, Co, and V, *T. cordata* showed intermediate values, whereas *T. tomentosa* generally exhibited the lowest extraction percentages. These results confirm that metal release into infusions varies markedly among elements and differs moderately among *Tilia* species.

The data presented in Tables S7–S9, which resolve individual brewing parameters, demonstrate that the transfer efficiency of heavy metals into Tilia flower infusions is primarily governed by water type, brewing temperature–time conditions, and species-specific physiological characteristics.

Across all three species—*Tilia tomentosa*, *Tilia cordata*, and *Tilia platyphyllos*—the results reveal a consistent and marked difference between infusions prepared with ultrapure water and those prepared with tap water. In samples brewed with tap water (INF-T6–T10, INF-C6–C10, and INF-P6–P10), metals such as Pb, Ni, Cr, Mn, and Al exhibited substantially higher transfer efficiencies, indicating that the mineral content and ionic strength of tap water greatly enhance the solubilization of metal ions from plant matrices.

As demonstrated by the condition-specific data in Tables S7–S9, temperature and infusion time represent a second major factor controlling metal release. Across all species, increasing the temperature from 70 °C to 95 °C and extending the brewing time from 5 to 15 min resulted in progressively higher transfer efficiencies, particularly for Zn, Cu, Mn, Ni, and Al. This thermally driven enhancement was especially pronounced in *Tilia cordata* (Table S8) and *Tilia platyphyllos* (Table S9), where the highest-temperature samples (INF-C10 and INF-P10) exhibited the most elevated values. These patterns confirm that metal extraction is strongly temperature-dependent, reflecting enhanced dissolution of metal-binding complexes and structural breakdown of plant tissues at higher thermal intensities.

Among the species, *Tilia cordata* (Table S8) generally showed the greatest metal transfer, especially for Ni, Zn, Mn, and Al, suggesting greater intrinsic mobility or weaker matrix binding of these elements within its tissues. *Tilia tomentosa* (Table S7) yielded moderate values overall but displayed notable increases in Pb and Ni under more extreme brewing conditions, while *Tilia platyphyllos* (Table S9) presented an intermediate profile, with particularly elevated Zn, Mn, and Cr release under specific conditions. These interspecific differences likely reflect variation in floral microstructure, metal compartmentalization, and storage strategies.

Taken together, the results from Tables S7–S9 emphasize that heavy metal transfer into *Tilia* infusions increases with higher temperature, longer infusion time, and the use of mineral-containing water, with considerable variation among species. These findings are critical for assessing the safety of herbal tea consumption, demonstrating that brewing conditions can significantly influence consumer exposure to heavy metals and that optimized preparation practices may help minimize potential toxicological risks.

### 2.8. Assessment of Contamination and Enrichment Indices in Urban and Forest Soils (CI, EF)

**Figure 10:**
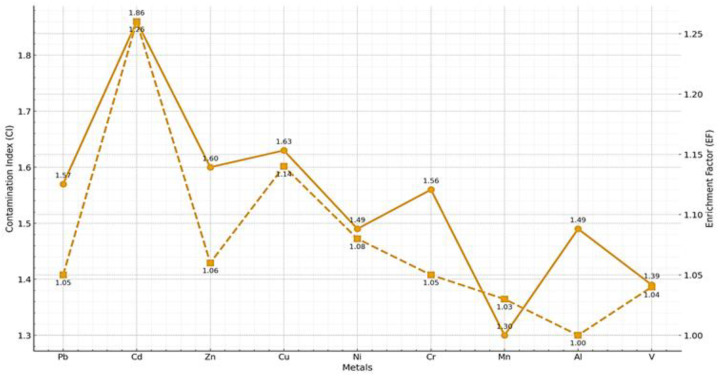
Dual-axis representation of Contamination Index (CI) and Enrichment Factor (EF) illustrating metal mobility and enrichment patterns in urban soils. Solid lines represent the Contamination Index (CI), while dashed lines represent the Enrichment Factor (EF).

Figure 10 provides an integrated representation of the Contamination Index (CI) and the Enrichment Factor (EF), enabling a more detailed evaluation of heavy metal accumulation and mobilization in urban soils relative to forest reference soils. The CI values, calculated as the ratio between urban and forest concentrations, indicate that most analyzed metals fall within a moderate contamination category. This is evident for elements such as Cd, Zn, Cu, Cr, Pb, and Ni, which exhibit CI values ranging from 1.49 to 1.86. The highest CI was recorded for cadmium, suggesting enhanced urban accumulation potentially influenced by sources such as road traffic, tire abrasion, and atmospheric deposition associated with combustion processes. In contrast, Mn and V displayed lower CI values, around 1.30–1.39, indicating slight contamination and reduced relative mobility in the urban context (Figure 10).

The interpretation of the EF, displayed on the secondary axis in Figure 10, clarifies the origin of the detected metals and highlights important distinctions between simple accumulation and true enrichment. Although the CI indicates a generalized increase in metal concentrations in urban soils, EF values reveal that most metals retain a predominantly lithogenic signature. The EF values for Pb, Zn, Ni, Cr, Mn, and V are very close to unity (1.03–1.08), indicating that these elements are not significantly enriched relative to aluminum, the geochemical reference element. This close correspondence between metal and Al behavior demonstrates that observed concentration differences are largely controlled by natural variations in soil parent material rather than by strong anthropogenic inputs.

Cadmium and copper constitute the only notable exceptions, exhibiting slightly elevated EF values of 1.26 and 1.14, respectively, indicative of a subtle yet detectable anthropogenic contribution. For cadmium, this mild enrichment is consistent with its elevated CI value and confirms that this element is particularly sensitive to urban influence, even in areas lacking major industrial activities. Copper, although showing a lower degree of enrichment, may be associated with sources such as infrastructure degradation, brake wear, and routine urban activities. Nevertheless, the overall low EF values indicate that enrichment remains moderate and does not reflect a significant geochemical alteration of the soil.

The combined interpretation of the CI and EF in Figure 10 emphasizes that urban soils accumulate metals to a moderate extent relative to forest soils, but without pronounced anthropogenic enrichment. This dissociation between absolute contamination (reflected by CI) and relative enrichment (reflected by EF) indicates that urbanization primarily increases metal concentrations via particle transport and atmospheric deposition, without inducing substantial changes in the geochemical baseline. Therefore, although an urban impact on the studied soils is evident, it remains moderate, and most metals are predominantly controlled by lithogenic sources. The clearest anthropogenic signals are associated with cadmium and copper; however, even in these cases, the levels remain low and do not indicate a high ecological risk.

### 2.9. Heatmap Comparison of Interspecific Metal Transfer Efficiency from Flowers to Infusions

**Figure 11:**
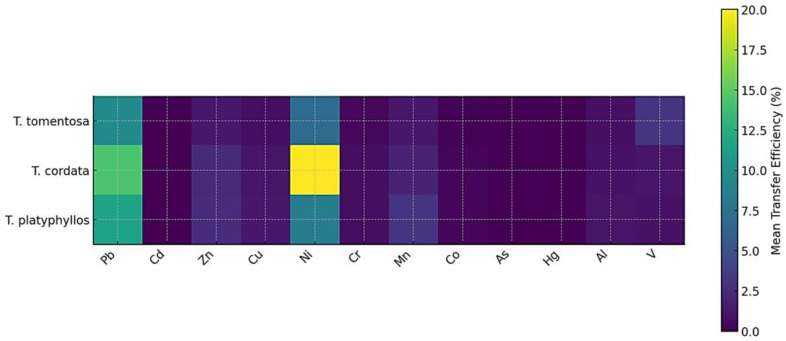
A comparative heatmap illustrating the mean transfer efficiency (%) of heavy metals from floral tissues into infusions across *Tilia tomentosa*, *Tilia cordata*, and *Tilia platyphyllos.*

As illustrated in Figure 11, the comparative heatmap reveals pronounced interspecific differences in the mean transfer efficiency of heavy metals into the final infusion stage. Although the heatmap explicitly represents only the terminal extraction step (flower → infusion), the observed patterns reflect the combined contribution of internal metal translocation along the soil–plant continuum and surface-associated metals retained on floral tissues, including atmospheric deposition. Because flowers were not pre-washed prior to analysis and infusion, surface-deposited atmospheric particles may have contributed to the metal concentrations measured in the infusions under realistic harvesting and infusion preparation conditions.

As shown in Figure 11, *Tilia cordata* exhibits the highest transfer efficiencies among the studied species, particularly for Ni, for which mean values exceed 20%. This pronounced signal indicates that Ni undergoes efficient uptake from soil, is readily translocated through bark and leaves, and ultimately accumulates in floral tissues in a highly soluble form. Such a pattern reflects enhanced physiological mobility and suggests that *T. cordata* possesses specific anatomical or biochemical traits—such as higher vascular conductivity, increased membrane permeability, or more effective chelation pathways—that facilitate metal movement along the plant continuum. The elevated values observed for Pb, Mn, and Al in *T. cordata* (Figure 8) further support the conclusion that this species mobilizes a broader spectrum of elements across all compartments, from soil to infusion.

In contrast, *Tilia tomentosa* exhibits a more conservative metal transfer profile. As shown in Figure 11, only Mn and Al display moderate extraction into the infusion, whereas elements such as Cd, Co, As, and Hg remain close to zero. This pattern suggests that *T. tomentosa* restricts metal translocation at earlier stages—either during root uptake or through bark retention—thereby limiting delivery to floral tissues. Consequently, the low intensities observed in the heatmap indicate that this species acts as a stronger biological barrier, effectively constraining metal migration along the soil → bark → leaf → flower continuum and ultimately reducing the solubility of these elements in the infusion.

*Tilia platyphyllos* exhibits an intermediate transfer behavior. As illustrated in Figure 11, Mn, Zn, and Al display modest but clearly discernible transfer efficiencies, indicating that these elements are moderately mobile through bark and leaves and reach floral tissues in extractable forms. Nevertheless, overall transfer values remain lower than those observed for *T. cordata*, suggesting the presence of more effective retention mechanisms within floral structures or reduced solubility during infusion preparation. This pattern is consistent with known metal storage strategies in certain species, which involve binding metals to structural polysaccharides or compartmentalizing them intracellularly, thereby limiting their final extractability.

Figure 11 clearly shows that metals such as Cd, Co, As, and Hg exhibit consistently low transfer efficiencies across all species, reflecting their limited bioavailability and strong binding affinity in both soils and plant tissues. In contrast, the higher transfer values observed for Ni, Mn, Al, and Zn highlight their greater mobility along the entire soil–plant continuum and their enhanced solubility during aqueous extraction from floral tissues. Collectively, these patterns underscore fundamental interspecific differences in metal uptake, translocation, and extractability into infusions, indicating that *T. cordata* exhibits the highest overall mobility, *T. platyphyllos* an intermediate profile, and *T. tomentosa* the most restricted metal movement from soil to final infusion.

### 2.10. Multivariate PCA of Species- and Brewing-Dependent Metal Mobilization in Tilia Infusions

**Figure 12:**
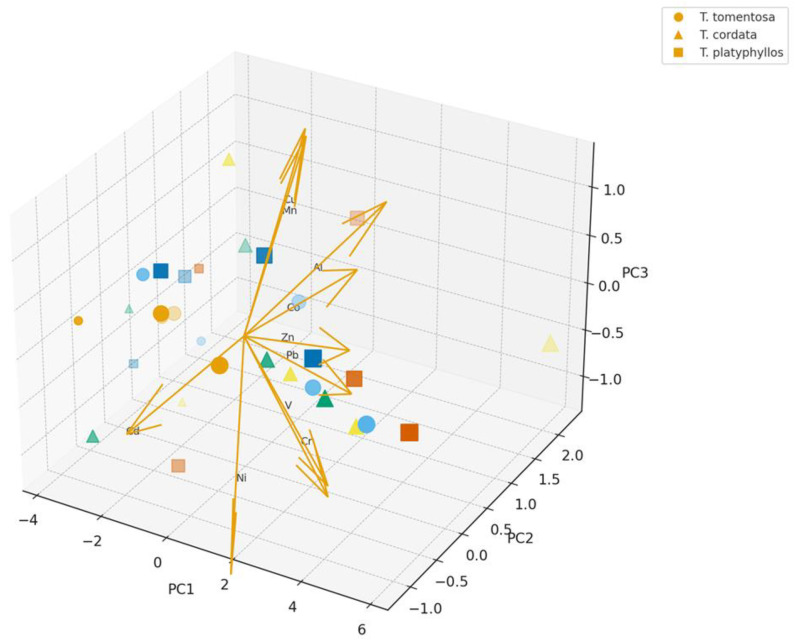
Three-dimensional PCA ordination of heavy metal profiles in *Tilia* flower infusions, showing element loadings and sample grouping according to species identity. Colors indicate species: *Tilia tomentosa*, *Tilia cordata*, and *Tilia platyphyllos*.

The multivariate PCA model presented in Figure 12 reveals a highly structured separation among *Tilia* flower infusions, demonstrating that brewing parameters—namely temperature, extraction time, and water type—interact strongly with species identity to control the mobilization of heavy metals into the aqueous phase. The clear spatial clustering of samples into distinct groups indicates that metal release behavior is not random, but rather follows species-specific and condition-dependent extraction patterns, providing insight into the physicochemical and morphological drivers governing trace metal solubility in herbal *Tilia* teas.

Figure 12 describes the effect of infusion temperature on total polyphenol content. Circles (●) represent samples prepared at 70 °C, triangles (▲) at 80 °C, and squares (■) at 90 °C. Data are expressed as mean ± SD (*n* = 3).

The distribution of samples along the PC1 and PC2 axes, which together account for the majority of total variance, highlights the dominant contribution of Mn, Zn, Cu, and Ni as major discriminating elements, as indicated by the direction and magnitude of the corresponding loading vectors in Figure 12. The pronounced loadings for Mn and Zn reflect their strong sensitivity to thermally enhanced extraction, confirming that higher brewing temperatures (90–95 °C) and the use of tap water accelerate leaching from floral structures. In contrast, elements such as Pb, Al, and V exhibit a more moderate contribution to sample separation, suggesting a closer association with particulate deposition on floral surfaces and slower desorption kinetics under typical infusion conditions. The short loadings observed for Cd, As, and Hg are consistent with detection-level behavior and their minimal statistical influence on the multivariate model.

Species-related clustering is particularly evident: *T. cordata* occupies the upper region of the PCA space associated with elevated concentrations of Mn, Zn, and Ni, whereas *T. platyphyllos* forms a transitional group characterized by intermediate metal mobility, and *T. tomentosa* tends toward lower-intensity clusters, except for occasional enrichment in Pb and V under high-temperature tap water brewing conditions. These patterns reinforce the experimental evidence indicating structural differences in floral microanatomy, glandular secretion characteristics, and particulate retention efficiency among species. The PCA thus corroborates the hierarchical extraction efficiency sequence observed in the experimental dataset.

Additionally, the clustering gradient strongly reflects the environmental history of the floral material, with infusions derived from urban samples positioned closest to the Mn–Zn–Cu vector directions, whereas those from forest habitats cluster in the opposite region of the ordination space, corresponding to low elemental mobility and low-pollution background conditions. This spatial distribution confirms the influence of atmospheric deposition and pollution intensity on the final infusion composition, underscoring the importance of harvest origin for ensuring herbal product safety.

From a public health and phytotherapeutic perspective, the PCA model demonstrates that both brewing conditions and botanical identity significantly modulate potential exposure pathways. Accordingly, controlling preparation parameters (e.g., moderate temperature and the use of purified water) and ensuring ethical sourcing (forest ecosystems versus urban roadsides) emerge as critical strategies for minimizing heavy metal transfer into consumed beverages. These findings further validate the utility of PCA as a powerful diagnostic tool for interpreting complex chemical–environment interactions in medicinal plants.

## 3. Discussion

In comparison with the concentration ranges reported for medicinal plant infusions worldwide, the results obtained in the present study for *Tilia* spp. infusions fall within the same order of magnitude or toward the lower end of published values. Thus, the Pb concentrations determined (0.23–0.31 µg/L) are comparable to or lower than those reported by Suchacz and Wesolowski (2012) [36] for various herbal infusions, as well as those reported by Kandić et al. (2023) [37] for medicinal plants consumed as teas, which generally remain below 1 µg/L.

Similarly, the concentrations of Ni (0.52–0.78 µg/L) and Cu (1.1–2.4 µg/L) fall within the lower ranges reported in recent international studies on medicinal plant infusions, including those presented by Akhbarizadeh et al. (2023) [38], who highlighted the wide variability in metal concentrations depending on plant species and geographical origin.

The levels obtained for Zn (6.4–13.4 µg/L) and Mn (18.6–33.1 µg/L) are also consistent with the typical ranges reported in the literature for herbal infusions, where Mn is frequently the dominant element and Zn generally occurs at moderate concentrations. Overall, comparison of the present results with those reported by Suchacz and Wesolowski (2012) [36], Kandić et al. (2023) [37], and Akhbarizadeh et al. (2023) [38] indicates that the metal concentrations measured in *Tilia* spp. infusions are not elevated and do not exceed the values commonly reported in international studies, confirming a contamination profile that is comparable to or lower than that observed in other medicinal plant infusions worldwide.

Urban vegetation is known to accumulate heavy metals as a result of atmospheric deposition and soil contamination, leading to measurable physiological changes in plant tissues, as demonstrated in several urban studies, including Doganlar et al. (2012) [39].

Tea and herbal infusion consumption in Romania is relatively low (~150–250 mL/day in adults) compared to the European Union average (300–400 mL/day), according to the EFSA Food Consumption Database and the national CARF study (2015–2020) (Neagu et al., 2020; EFSA Food Consumption Database) [40,41]. In countries with a strong tea tradition (e.g., the UK and the Netherlands), daily intake may exceed 600–800 mL/day (EFSA Food Consumption Database) [41]. Consumption patterns vary by age and sex, with generally lower intake observed among children.

The EFSA further confirms that herbal teas constitute a major dietary source of certain natural contaminants, such as pyrrolizidine alkaloids (EFSA Scientific Committee, 2012) [42]. The Romanian Dietary Guidelines recommend a total daily fluid intake of 2–2.5 L, including unsweetened teas (WHO/FAO, 2009) [43].

Accordingly, in exposure assessment scenarios for metal intake through tea consumption, indicative thresholds of 150 mL/day (low), 250–400 mL/day (moderate), and over 500 mL/day (high) are commonly applied, in line with EFSA exposure assessment models and default assumptions (EFSA Scientific Committee, 2012) [42].

Both the European Food Safety Authority (EFSA) and the World Health Organization (WHO) apply standardized default body weights in food risk assessment frameworks. The EFSA has updated the default adult body weight from 60 kg to 70 kg in order to better reflect the current European population (EFSA Scientific Committee, 2012) [42].

For children, recommended default values range from approximately 12 kg (1–3 years) to 22–23 kg (4–10 years), 42–43 kg (10–14 years), and 56–57 kg (14–17 years), while sex-specific differences are observed in adults (approximately 66 kg for women and 75–78 kg for men) [44]. Among elderly populations, average body weights generally range between 65 and 75 kg.

The WHO continues to apply the precautionary default value of 60 kg for adults in certain assessments, while explicitly acknowledging demographic variability (WHO/FAO, 2009) [43]. In the absence of locally derived biometric data, these default values are widely used in international dietary exposure assessments for food contaminants.

Figure 10 provides an overview of the Estimated Daily Intake (EDI) of twelve heavy and trace metals (Pb, Cd, Zn, Cu, Ni, Cr, Mn, Co, As, Hg, Al, and V) mobilized into *Tilia* flower infusions, differentiated by age group and consumption scenario (150 vs. 400 mL/day). The multi-panel layout enables a simultaneous comparison of age-related, metal-specific, and scenario-dependent variability, thereby offering an integrated assessment of potential dietary exposure associated with linden infusion consumption.

For all metals detected in the infusions (Pb, Cd, Zn, Cu, Ni, Cr, Mn, Co, Al, and V), young children (1–3 years) represent the most vulnerable group, followed by children aged 4–10 years. This pattern reflects their lower body mass relative to intake volume, resulting in EDI values up to 5–10 times higher than those observed in adults. The effect is particularly pronounced for Mn, Al, Zn, and Ni, for which the highest EDI values occur in the 1–3-year age group under the higher consumption scenario (400 mL/day).

For essential metals (Zn, Cu, and Mn), the observed distributions reflect both their high physiological mobility in plant tissues and their efficient extraction into hot water. Manganese (Mn) emerges as the dominant contributor to EDI, reaching values above 7 µg/kg/day in the 1–3-year age group. Although Mn is an essential micronutrient, excessive intake in children has been associated with neurotoxic effects, underscoring the importance of monitoring consumption patterns.

Among toxic non-essential metals, Pb and Cd exhibit pronounced increases with higher consumption, with EDI values in young children rising approximately threefold from low- to medium-intake scenarios. While absolute levels remain below EFSA-established adult TDI values, the substantially higher relative exposure in children warrants caution, particularly in the context of frequent consumption.

For Co, V, and Al, EDI values are moderate; however, their toxicological relevance depends on element-specific reference doses and bioavailability. Aluminum (Al) shows relatively elevated EDI values, comparable to those of some essential metals, reflecting its high extractability under hot water conditions. Given Al’s association with neuroinflammatory processes, its contribution via linden infusions may be relevant in cumulative exposure scenarios.

In contrast, As and Hg were below detection limits in all samples, resulting in negligible EDI across all age groups, which indicates minimal contamination and low extractability into herbal infusions—an encouraging outcome for consumer safety.

**Figure 13:**
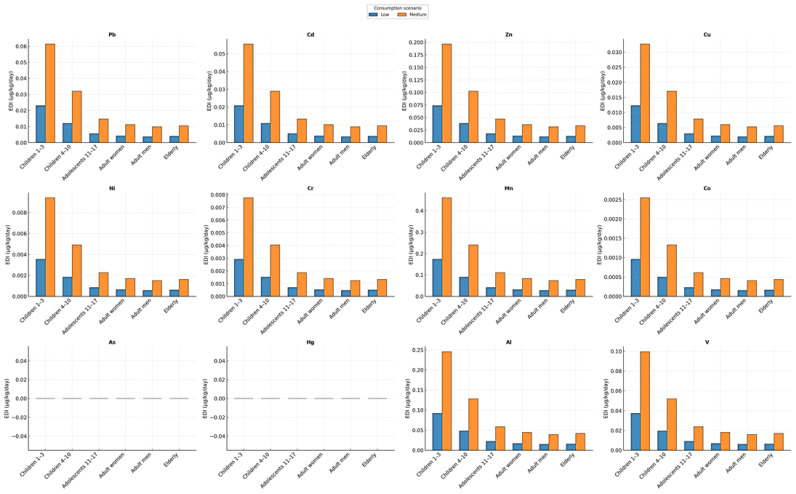
Estimated daily intake (EDI) of heavy and trace metals from *Tilia* infusions by age group and consumption scenario.

Comparisons among adult groups reveal minimal differences between women, men, and the elderly, largely attributable to variations in body weight. EDI values decrease progressively as body mass increases, resulting in the lowest relative exposure being observed in adults. Figure 13 clearly demonstrates that exposure risk is driven predominantly by age and consumption volume, rather than by *Tilia* species or metal type alone. The highest EDI values occur in young children under moderate-consumption scenarios, underscoring the need for prudent recommendations regarding frequent (daily) intake of linden flower infusions in this age group.

The comparative analysis of Estimated Daily Intake (EDI) across age groups demonstrates that body weight and consumption volume are the primary determinants of exposure, rather than metal type alone. Young children (1–3 years), for whom the EFSA assigns a default body weight of 12 kg, experience the highest exposure, with EDI values up to 5–10 times higher than those observed in adults (66–78 kg) at equivalent consumption volumes (150–400 mL/day). Under the moderate consumption scenario (400 mL/day), nearly all metals reach their maximum EDI in this youngest group, whereas adult exposure remains minimal, consistent with typical Romanian consumption patterns (150–250 mL/day). This inverse relationship between body mass and dose per kilogram underpins the interpretation of exposure-related risk across all analyzed elements.

When EDI values are compared with EFSA/JECFA toxicological reference thresholds, exposure from *Tilia* infusions is found to be well below health-based guidance values for most metals. Essential elements such as Zn and Cu exhibit very low EDI values (<1 µg/kg/day), far below their respective limits of 360 µg/kg/day (UL for Zn) and 70 µg/kg/day (ADI for Cu).

Manganese—the element with the highest extraction efficiency—reaches approximately 7 µg/kg/day in children aged 1–3 years under the 400 mL/day scenario, representing only ~4–5% of the EFSA/ATSDR reference value of 160 µg/kg/day.

Toxic metals also display substantial safety margins: Pb and Cd remain below 10–15% of their critical values (0.5 µg/kg/day for Pb, 0.36 µg/kg/day for Cd), even in the most sensitive group. Exposure to Ni (TDI = 2.8 µg/kg/day), Cr(III) (TDI = 300 µg/kg/day), and Co (HBGV = 1.6 µg/kg/day) is several orders of magnitude lower than their respective thresholds.

Aluminum shows moderate extractability; however, EDI values remain only a few percent of the TWI (140 µg/kg/day). For vanadium, EDI values are well below the broad proposed HBGV range (0.07–26 µg/kg/day), indicating limited toxicological relevance at the concentrations observed.

Overall, the comparison between EDI values and health-based reference limits demonstrates that *Tilia* infusions contribute minimally to dietary metal exposure at realistic consumption levels in Romania. Only manganese—and to a lesser extent aluminum, lead, and cadmium—warrants contextual attention in young children consuming the infusion daily, where low body weight amplifies the dose per kilogram.

The absence of arsenic and mercury in all samples (EDI ≈ 0), despite their stringent PTWI values (2.1 µg/kg/day for inorganic As and 0.23 µg/kg/day for methylmercury), represents a major safety advantage.

In summary, *Tilia* flower tea can be considered a safe beverage with respect to metal content, provided consumption remains age-appropriate, integrated into a varied diet, and exposure remains well below EFSA toxicological reference levels.

A ratio-based interpretation of the results indicates that all metals mobilized into *Tilia* infusions remain far below their respective toxicological thresholds, with EDI-to-reference ratios generally <0.05 across all age groups. The highest relative contributions are observed for Mn, Pb, Cd, and Al in children aged 1–3 years; however, even in this most sensitive category, the ratios do not exceed 0.10–0.15 of the corresponding guidance values.

All other elements, including Zn, Cu, Ni, Cr, Co, and V, remain at trace proportions (<0.01), indicating negligible toxicological relevance. These ratio patterns confirm that the variability observed across species, habitats, and extraction conditions has minimal impact on overall health risk, and that the safety profile of *Tilia* infusions is maintained across plausible consumption scenarios.

Several limitations should be considered when interpreting the results of this study. First, the investigation was conducted within a single geographic region; therefore, local environmental and geochemical conditions may constrain the direct extrapolation of the findings to other urban or peri-urban contexts. In addition, sampling was performed during a single season, which does not capture potential temporal variability in metal deposition, uptake, and translocation.

While the applied contamination and enrichment indices (CI and EF) provide reliable insights into accumulation patterns, they are based on total metal concentrations and do not account for metal speciation or bioavailability. Furthermore, the assessment of metal transfer into infusions and the associated exposure estimates relied on standardized preparation conditions and consumption assumptions which, although widely accepted, may not fully reflect individual brewing practices or dietary habits.

These limitations highlight the need for future studies incorporating multi-seasonal datasets, broader spatial coverage, speciation analyses, and more refined exposure scenarios. It should be emphasized that brewing temperature and infusion time were applied as paired parameters; therefore, their individual effects cannot be interpreted independently. Because temperature and infusion time were applied as paired parameters, the present design does not allow for the identification of an optimal infusion time or temperature as independent variables.

## 4. Materials and Methods

### 4.1. Study Area and Sampling Design

The study area is located in north-western Romania, within Bihor County, a region characterized by a pronounced geomorphological gradient ranging from the low western plains to the hilly and mountainous areas of the Apuseni Mountains, with elevations spanning approximately 80 to 1800 m.

Sampling sites were classified into three habitat categories (urban, local, and forest) based on operational criteria reflecting the degree of anthropogenic pressure. Urban sites were located within the administrative boundaries of the city of Oradea and were characterized by high population density (approximately 2000–3000 inhabitants/km^2^), intense road traffic along major arteries (>10,000–15,000 vehicles/day), and continuous built-up infrastructure. Local sites corresponded to peri-urban and rural settlements surrounding the city, with intermediate population density (approximately 300–800 inhabitants/km^2^), moderate traffic intensity (approximately 1000–5000 vehicles/day), and mixed land use (residential–agricultural). Forest sites were selected outside residential areas, at a distance from heavily trafficked roads, with no direct traffic influence and minimal anthropogenic disturbance, and were considered representative of regional geochemical background conditions.

This marked variability in relief and soil conditions, combined with contrasting land use patterns, provides an appropriate framework for assessing the influence of both natural and anthropogenic factors on heavy metal accumulation in *Tilia* species. The sampling design was developed to capture a well-defined urban–local–forest gradient, centered on the Oradea metropolitan area and extending toward peri-urban and forested zones with progressively reduced anthropogenic impact.

**Figure 14:**
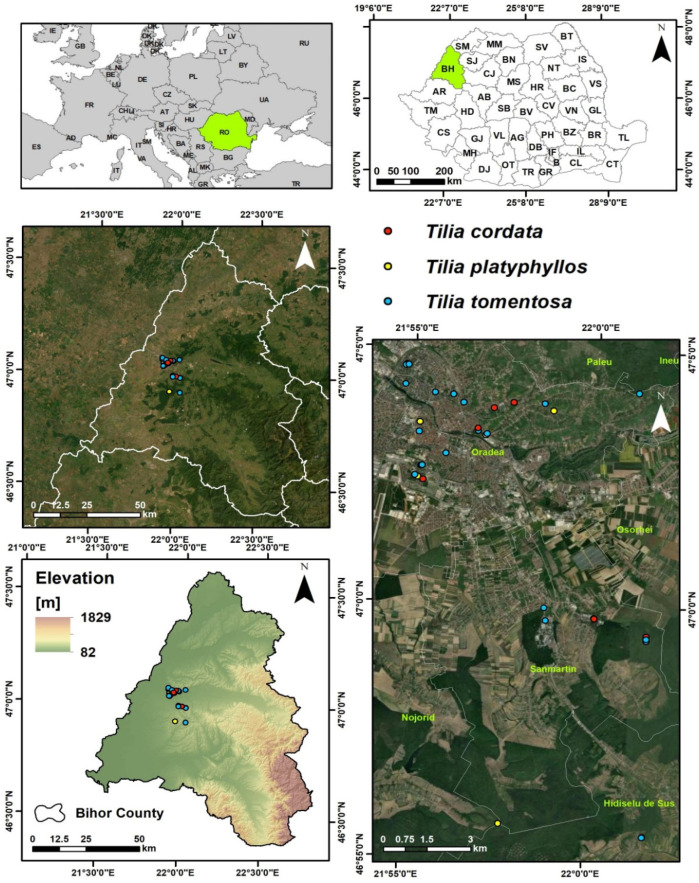
The location of the study area and spatial distribution of sampling sites for *Tilia* species in Bihor County (north-western Romania).

Representative sites were selected for three common species in the region (*Tilia cordata*, *Tilia platyphyllos*, and *Tilia tomentosa*), distributed across urban green spaces, rural settings, and forest environments, thereby allowing for direct comparison of contamination levels as a function of habitat type and species. Figure 14 summarizes the geographic position of the study area at both regional and national scales, together with administrative boundaries, altitudinal framework, and the spatial distribution of sampling sites.

Sampling points were evenly distributed across the county, thereby avoiding excessive clustering within a single environmental category. Urban sites reflect areas subjected to high anthropogenic pressure (traffic and residential activities), local sites indicate moderate impact, while forest sites are considered representative of the regional geochemical background.

Through this strategy, the study area and sampling design provide a robust basis for interpreting the spatial distribution of heavy metals and for evaluating their transfer along the soil–plant–infusion pathway, thereby supporting the use of *Tilia* species as effective bioindicators along the urban–forest gradient. Moreover, this approach enables a consistent comparison between habitat types and species-specific responses, strengthening the assessment of anthropogenic influences on metal accumulation patterns.

The number of sampled trees was determined based on field feasibility and the availability of mature and healthy *Tilia* individuals that allowed for the collection of a complete set of matrices from the same location. Each tree was treated as an independent experimental unit, resulting in a total of 34 trees included in the study. In one case, two trees were located in close spatial proximity and shared the same edaphic context; therefore, a single representative soil sample was collected for both trees. Consequently, soil analyses were performed on a total of 33 samples.

**Figure 15:**
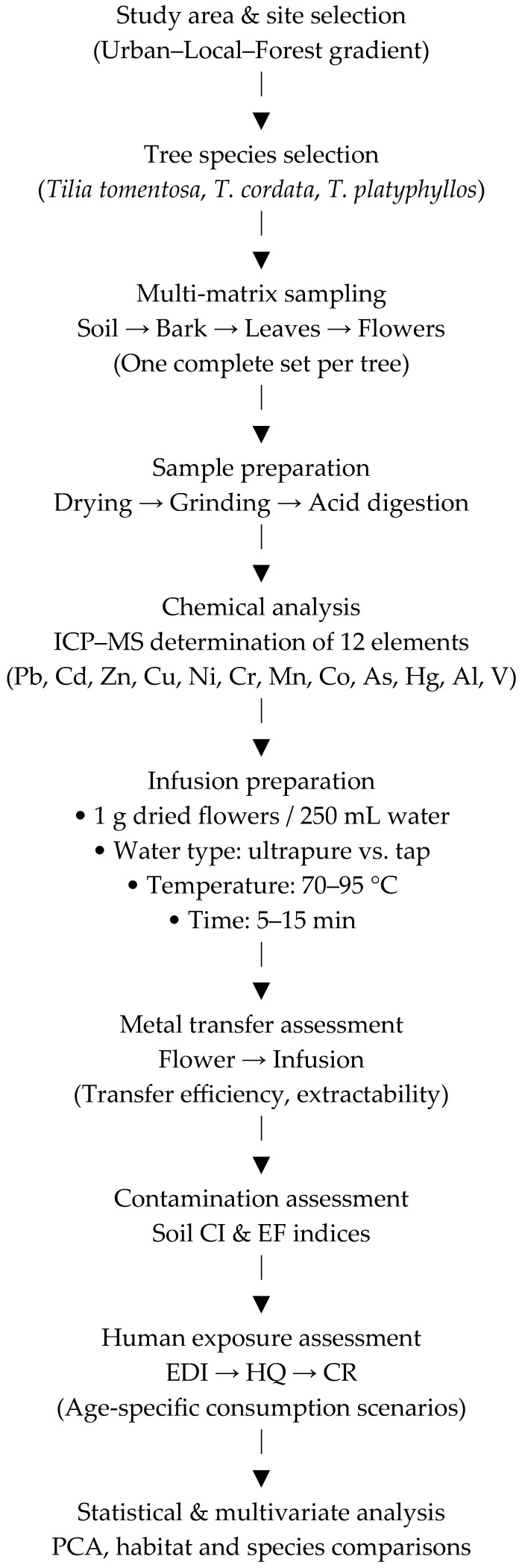
Schematic Overview of Experimental Design and Analytical Workflow.

An overview of the experimental design and analytical workflow is presented in Figure 15. The schematic summarizes the main methodological steps of the study, including site selection along an urban–forest gradient, *Tilia* species selection, multi-matrix sampling (soil, bark, leaves, and flowers), sample preparation and elemental analysis by ICP–MS, followed by infusion preparation under controlled conditions. The workflow further illustrates the assessment of metal transfer from plant material to infusions, soil contamination indices, human exposure and risk evaluation, and the applied statistical and multivariate analyses.

### 4.2. Sample Collection and Preparation

The study included 34 *Tilia* trees, from which a total of 136 samples were collected: 34 soil, 34 bark, 34 leaf, and 34 flower samples (one complete set per tree). Soil samples were collected from the rhizosphere (0–10 cm depth) beneath the canopy. Five subsamples per tree, collected at distances of 0.5–1 m from the trunk, were combined into one composite sample (~500 g).

Bark samples (5–10 g) were taken from the outer trunk layer at a height of approximately 1.3 m, avoiding damaged or colonized areas. Fully developed, healthy leaves were collected from comparable canopy positions, while intact inflorescences were harvested during peak flowering. All samples corresponding to each tree originated from the same site in order to ensure comparability across matrices.

Sampling was conducted under dry conditions, avoiding rainfall within 48 h prior to collection. Disposable nitrile gloves were worn and changed between samples. All tools were made of stainless steel or inert plastic and were cleaned with deionized water and 70% ethanol between uses.

In the laboratory, plant samples were oven-dried at 40 °C to constant weight, while soil samples were air-dried at room temperature. Soil samples were subsequently homogenized and sieved to <2 mm. Dried plant material was ground using a stainless-steel mill and sieved to <0.5 mm. All samples were stored in airtight polyethylene or polypropylene containers in a cool, dry, and dark environment until analysis.

Strict contamination control procedures were applied throughout sampling, preparation, and storage to ensure analytical reliability. Detailed information on species identification, sampling locations, geographic coordinates, site characteristics, sampling dates, and the correspondence between individual *Tilia* trees and their associated soil, bark, leaf, and flower samples is provided in Table S10.

### 4.3. Physicochemical Characterization of Soils

Soil physicochemical properties were determined on air-dried, homogenized samples sieved to <2 mm. Soil pH was measured potentiometrically in distilled water and 1 M KCl using a bench-top digital pH meter equipped with a combined glass electrode (Metrohm AG, Herisau, Switzerland), at a soil-to-solution ratio of 1:2.5 (*w*/*v*), following ISO 10390. Electrical conductivity (EC) was determined in a 1:5 (*w*/*v*) soil–water extract using a digital conductivity meter (WTW GmbH, Weilheim, Germany), in accordance with ISO 11265. All pH and EC measurements were performed at 20 ± 1 °C.

Soil texture (sand, silt, and clay fractions) was determined by the pipette/hydrometer method after dispersion with sodium hexametaphosphate (Suprapur^®^, Merck KGaA, Darmstadt, Germany), following ISO 11277. Particle size distribution was subsequently used to define soil textural classes and to support the interpretation of soil physical constraints.

Soil organic carbon (C_org) was determined by the Walkley–Black dichromate oxidation method according to ISO 14235, using potassium dichromate and concentrated sulfuric acid (Suprapur^®^, Merck KGaA, Darmstadt, Germany). Total nitrogen (N_total) was quantified using the Kjeldahl digestion–distillation method (VELP Scientifica S.r.L., Usmate Velate, Italy), following ISO 11261, employing Kjeldahl catalyst tablets, sodium hydroxide, boric acid, and standard titration solutions (Suprapur^®^/Titrisol^®^, Merck KGaA, Darmstadt, Germany). The C/N ratio was calculated from the measured C_org and N_total values. The applied methodology is suitable for non-carbonate or weakly calcareous soils and provides a reliable estimate of soil organic matter content.

All analyses were performed in duplicate, and analytical quality was assured through routine calibration of instruments, the use of analytical-grade reagents, and the inclusion of procedural blanks. Analytical precision was within ±5% for all measured parameters, and results were expressed on a dry-weight basis. Supplementary Table S11 summarizes additional details related to the soil physicochemical analyses. Variations in soil organic carbon and C/N ratio observed in the present study are consistent with patterns reported for organic and conventional systems in Romanian soils [45].

### 4.4. Determination of Heavy Metals in Solid Matrices

The determination of heavy metals in solid matrices (soil, bark, leaves, and flowers) was carried out following complete acid digestion of dried and homogenized samples. Approximately 0.25–0.50 g of each sample was accurately weighed using an analytical balance (Mettler-Toledo GmbH, Greifensee, Switzerland) and transferred into closed polytetrafluoroethylene (PTFE) digestion vessels.

Microwave-assisted acid digestion was performed using a high-pressure microwave system (Milestone S.r.L., Sorisole, Italy). Samples were digested with concentrated HNO_3_ (65%) and H_2_O_2_ (30%) (Suprapur^®^, Merck KGaA, Darmstadt, Germany). Matrix-specific digestion programs were applied depending on sample type (Table S12). After cooling, the digests were quantitatively transferred and diluted to a known final volume with ultrapure water (Milli-Q^®^, Merck, Darmstadt, Germany). Procedural blanks were prepared and treated following the same protocol.

Elemental analysis was performed using quadrupole inductively coupled plasma mass spectrometry (ICP–MS; Thermo Scientific iCAP Q, Darmstadt, Germany). The instrument was operated under optimized conditions, employing helium collision mode to minimize polyatomic and matrix-related interferences. Instrumental operating conditions and data acquisition parameters are summarized in Table S13.

Calibration was performed using multi-element standard solutions prepared from certified stock materials (Certipur^®^, Merck KGaA, Darmstadt, Germany) in 1% (*v*/*v*) HNO_3_ (Suprapur^®^, Merck). Internal standards (Rh, In, and Re) were applied to correct for instrumental drift and matrix effects. Calibration curves exhibited excellent linearity, with coefficients of determination (R^2^) ranging between 0.9996 and 0.9999.

Limits of detection (LOD) and quantification (LOQ) were determined from procedural blanks (Table S14). Lead (Pb), cadmium (Cd), zinc (Zn), copper (Cu), nickel (Ni), chromium (Cr), manganese (Mn), cobalt (Co), arsenic (As), mercury (Hg), aluminum (Al), and vanadium (V) were quantified in all investigated matrices.

Analytical accuracy and precision were verified using certified reference materials and recovery tests, yielding recoveries between 90 and 110% and relative standard deviations (RSD) below 5%. The applied digestion and ICP–MS analytical approach is consistent with methodologies previously reported for the determination of heavy metals in soils and plant matrices from north-western Romania [46,47,48,49].

### 4.5. Preparation of Tilia Flower Infusions

*Tilia* flower infusions were prepared using 1.00 g ± 0.01 g of dried and finely ground plant material, to which 250 mL of water was added. Two types of water were employed, ultrapure water (Milli-Q^®^) and tap water, in order to simulate both controlled laboratory conditions and realistic domestic preparation practices.

The water was heated to temperatures ranging between 70 and 95 °C and subsequently poured over the plant material. Water temperature was measured immediately before pouring using a calibrated Hanna Instruments HI93509 Checktemp^®^ digital thermometer with stainless steel immersion probe (Hanna Instruments, Woonsocket, RI, USA) to ensure accurate temperature control. Heating was carried out on a Velp AREC 7 digital ceramic hot plate (Velp Scientifica, Usmate Velate, MB, Italy) equipped with electronic temperature regulation to achieve stable target temperatures prior to infusion.

Infusion times ranged between 5 and 15 min, according to the predefined experimental design. Temperature and infusion time were intentionally applied as paired temperature–time combinations defining two standardized brewing scenarios (mild extraction: 70 °C/5 min; intensive extraction: 95 °C/15 min), representative of common domestic tea preparation practices; therefore, they were not treated as independent experimental variables. Accordingly, the results reported in Table S6 and discussed throughout the manuscript reflect the combined effect of brewing temperature and infusion duration on metal mobilization.

After infusion, samples were allowed to cool to room temperature and were filtered to remove solid residues. The resulting infusions were collected in pre-cleaned polypropylene containers and stored at 4 °C until heavy metal analysis by ICP–MS.

All infusions were prepared in triplicate, and procedural blanks were processed using identical water volumes without plant material to control for potential background contamination. The experimental conditions applied for the preparation of *Tilia* flower infusions, including plant material mass, water type, infusion temperature and time, sample handling, and quality control procedures, are summarized in Table S15.

### 4.6. Quality Assurance and Quality Control (QA/QC)

Analytical accuracy and precision were evaluated through the analysis of certified reference materials and recovery tests. Method validation was performed using SRM 2709a (San Joaquin Soil) for soil samples (Table S16), NMIJ CRM 7505-a (Tea Leaves) for plant material (Table S17), and NIST SRM 1643f (Trace Elements in Water) for aqueous samples and infusions (Table S18). Recoveries were generally within the range of 90–110%, and relative standard deviations were below 5%, confirming the robustness and reproducibility of the analytical procedure across all investigated matrices.

### 4.7. Contamination and Enrichment Indices

For the calculation of contamination and enrichment indices, background values were defined based on site-specific baseline conditions derived from forest samples collected within the present study. Specifically, background concentrations were calculated as the mean elemental levels measured in forest reference samples, selected to represent local natural geochemical conditions with minimal anthropogenic influence.

Contamination and enrichment indices, such as the contamination factor (CF) and enrichment factor (EF), are widely applied tools for evaluating heavy metal accumulation in soils and for distinguishing between natural and anthropogenic contamination sources, as demonstrated in previous environmental studies [50,51,52]. To assess the degree of metal contamination and the influence of anthropogenic inputs, contamination and enrichment indices were calculated using measured metal concentrations in soil samples associated with *Tilia* species.

The contamination factor (CF) for each element was calculated according to Equation (1):

(1) $$CF=\frac{C_{sample}}{C_{background}}$$

where $C_{sample}$ is the concentration of the element measured in the soil sample (mg kg^−1^, dry weight), and $C_{background}$ is the corresponding background concentration, defined as the mean value measured in forest soils within the study area and considered representative of the regional geochemical background. Contamination levels were classified as low (CF < 1), moderate (1 ≤ CF < 3), considerable (3 ≤ CF < 6), and very high (CF ≥ 6).

The enrichment factor (EF) was used to distinguish between natural and anthropogenic sources of metals and was calculated according to Equation (2):

(2) $$EF=\frac{(C_{metal}/C_{Al}{)}_{sample}}{(C_{metal}/C_{Al}{)}_{background}}$$

where $C_{metal}$ and $C_{Al}$ are the concentrations (mg kg^−1^, dry weight) of the investigated metal and aluminum, respectively, in the soil sample and in the background soil. Aluminum was selected as the reference element due to its lithogenic origin, high natural abundance, and low susceptibility to anthropogenic enrichment. EF values were interpreted as follows: EF < 2 (minimal or no enrichment), 2–5 (moderate enrichment), 5–20 (significant enrichment), 20–40 (very high enrichment), and >40 (extreme enrichment).

All CF and EF values were calculated on a dry-weight basis using mean background concentrations derived from forest soils within the study area. These indices provided a quantitative framework for evaluating metal accumulation intensity and identifying potential anthropogenic contributions along the urban–forest gradient.

### 4.8. Human Health Risk Assessment

The human health risk assessment associated with the consumption of medicinal plant infusions was based on the standardized models described by Alawadhi et al. (2024) [4], which consider oral ingestion as the primary exposure pathway. The estimated daily intake was calculated by integrating metal concentrations with consumption volumes and body weights specific to different age groups, while non-carcinogenic and carcinogenic risks were assessed by relating EDI values to reference doses established by international regulatory bodies [4].

The estimated daily intake (EDI) of each metal was calculated according to Equation (3):

(3) $$EDI=\frac{C_{infusion}\times IR}{BW}$$

where $C_{infusion}$ is the concentration of the metal measured in the infusion (µg L^−1^), IR is the ingestion rate of the infusion (L day^−1^), and BW is the body weight of the exposed individual (kg). Two realistic consumption scenarios were considered: 150 mL day^−1^ (average intake) and 400 mL day^−1^ (high intake), consistent with common consumption patterns. Body-weight values specific to different age groups (children and adults) were adopted according to EFSA default exposure parameters.

The non-carcinogenic risk associated with each metal was evaluated using the hazard quotient (HQ), calculated as follows:

(4) $$HQ=\frac{EDI}{RfD}$$

where RfD is the oral reference dose (µg kg^−1^ day^−1^) established by international regulatory agencies (EFSA, USEPA, or JECFA). An HQ value < 1 indicates no significant carcinogenic risk, whereas HQ ≥ 1 suggests potential health concern. The overall non-carcinogenic risk posed by multiple metals was expressed as the hazard index (HI), calculated as the sum of individual HQ values for all analyzed elements.

For metals classified as carcinogenic via oral exposure, the carcinogenic risk (CR) was estimated using Equation (5):

(5) CR=EDI×SF

where SF is the oral cancer slope factor ((mg kg^−1^ day^−1^)^−1^). CR values were interpreted according to internationally accepted thresholds, with values below 10^−6^ considered negligible and values above 10^−4^ indicating unacceptable risk.

All risk calculations were performed using mean metal concentrations measured in infusions, expressed on a wet-weight basis, and the results were evaluated separately for each age group and consumption scenario. This approach provided a conservative and realistic assessment of potential health risks associated with the consumption of *Tilia* herbal infusions under typical and high-intake conditions.

### 4.9. Statistical Analysis

Statistical analyses were conducted to assess differences in metal concentrations among matrices (soil, bark, leaves, flowers, and infusions), *Tilia* species (*T. tomentosa*, *T. cordata*, *T. platyphyllos*), and habitat types (urban, local, and forest). Data normality was tested using the Shapiro–Wilk test. As most variables were non-normally distributed, non-parametric methods were applied. Group differences were evaluated using the Kruskal–Wallis test followed by Dunn’s post hoc test with Bonferroni correction. Relationships between metal concentrations and soil physicochemical properties were examined using Spearman’s rank correlation. Multivariate patterns were explored using principal component analysis (PCA) on z-score standardized variables. All analyses were performed in R (v4.3.2), and statistical significance was set at *p* < 0.05.

### 4.10. Ethical Considerations

This study did not involve human participants, personal data, clinical interventions, or experimental animals. All samples consisted exclusively of environmental materials (soil and plant tissues) collected from public or non-protected areas, in compliance with local regulations. No protected or endangered species were involved, and no specific permits or ethical approvals were required.

The study was conducted in accordance with institutional guidelines for environmental research and data integrity. Language editing and minor stylistic refinements were supported by automated tools. All scientific content, including study design, data analysis, interpretation of results, and conclusions, was developed exclusively by the authors, who assume full responsibility for the integrity and originality of the work. The preparation of the manuscript adhered to the journal’s policies on transparency and responsible research conduct.

## 5. Conclusions

This study demonstrates that *Tilia* spp. effectively capture and reflect heavy metal distribution patterns along urban–forest gradients, exhibiting a clear multi-matrix transfer pathway from soil and bark to leaves, flowers, and ultimately herbal infusions. Although certain elements—particularly Mn, Al, Pb, and Cd—show higher extractability and increased estimated daily intake (EDI) in young children due to their lower body weight, all calculated exposures remain well below the toxicological reference values established by the EFSA and JECFA.

The absence of As and Hg in all infusions represents a significant safety advantage. Overall, *Tilia* infusions contribute only marginally to total dietary metal exposure under realistic consumption scenarios, indicating that linden tea can be considered safe for the general population when consumed in moderate quantities.

Beyond the food safety perspective, the multi-matrix approach applied in this study confirms the utility of *Tilia* species as sensitive bioindicators of airborne and soil-derived metal inputs in complex urban–forest environments, supporting their use in integrated environmental monitoring and risk assessment frameworks.

## Data Availability

The data presented in this study are available from the corresponding author upon reasonable request.

## Supplementary Materials

The following supporting information can be downloaded at: https://www.mdpi.com/article/10.3390/ijms27041856/s1.

## Abbreviations

The following abbreviations are used in this manuscript:

Al	Aluminum
As	Arsenic
BD	Bulk density
BEC	Background equivalent concentration
BLD	Below limit of detection
Caex	Exchangeable calcium
CAT	Catalase activity
Cd	Cadmium
CF	Contamination factor
Co	Cobalt
Corg	Organic carbon
CR	Carcinogenic risk
Cr	Chromium
DHG	Dehydrogenase activity
EC	Electrical conductivity
EDI	Estimated daily intake
EF	Enrichment factor
EFSA	European Food Safety Authority
GM	Gravimetric moisture
Hg	Mercury
HI	Hazard index
HQ	Hazard quotient
HUM	Humus content
ICP–MS	Inductively coupled plasma mass spectrometry
ISO	International Organization for Standardization
JECFA	Joint FAO/WHO Expert Committee on Food Additives
Kex	Exchangeable potassium
LoD	Limit of detection
LoQ	Limit of quantification
Mgex	Exchangeable magnesium
Mn	Manganese
MR	Microbial respiration
Ni	Nickel
Nt	Total nitrogen
Pavail	Plant-available phosphorus
PCA	Principal component analysis
PHO	Phosphatase activity
Pb	Lead
PTFE	Polytetrafluoroethylene
QA/QC	Quality assurance/quality control
RSD	Relative standard deviation
SRM	Standard reference material
TLA	Three-letter acronym
TP	Total porosity
USEPA	United States Environmental Protection Agency
V	Vanadium
WHC	Water-holding capacity
Zn	Zinc
