# Supplementary material for: Molecular and Environmental Elucidation of Heavy Metal Transfer in Tilia spp.: From Soil Systems to Herbal Infusions Across Urban–Forest Gradients
Source: Int J Mol Sci. 2026 Feb 14;27(4):1856. doi: 10.3390/ijms27041856 (PMC12940536; doi:10.3390/ijms27041856)
Supplement: Supplementary file 1 [file ijms-27-01856-s001.zip › ijms-4136920-supplementary.pdf]

International Journal of *Molecular Sciences*

Table S1a. Physical and Chemical Properties of Soil Samples Corresponding to Tilia Trees

Identification Code	BD (g/cm^3)	TP (%)	GM (%)	WHC (%)	Sand (%)	Silt (%)	Clay (%)	pH (H_2O)	pH (KCl)	EC (µS/cm)	Corg (%)	Nt (%)	C/N (-)	Pavail (mg/kg)	Kex (mg/kg)	Caex (cmol(+)/kg)	Mgex (cmol(+)/kg)
TL-S01	1.04	60	15.49	42.77	42.47	34.9	22.63	6.66	5.95	187.6	2.78	0.2	14.04	28.74	109.92	8.74	2.02
TL-S02	1.43	45.87	12.29	29.89	55.32	32.92	11.75	7.79	7.25	427.82	1.03	0.14	7.52	30.23	197.72	17.39	2.92
TL-S03	1.38	47.79	14.93	32.7	52.19	36.97	10.84	7.64	7.27	528.07	1.6	0.12	13.73	22.51	155.61	12.59	2.55
TL-S04	1.42	46.55	14.65	35.52	57.11	36.35	6.54	8.1	7.47	346.1	1.69	0.05	20	26.4	128.62	15.49	3.1
TL-S05	1.5	43.56	11.35	29.82	46.18	20.43	33.38	7.71	7.23	469.02	1.55	0.09	17.09	47.98	165.57	12.73	2.94
TL-S06	1.36	48.7	12.49	39.8	46.44	35.9	17.66	7.51	6.78	291.28	0.9	0.11	8.05	19.27	189.55	14.71	2.58
TL-S07	1.61	39.24	13.04	25.86	51.79	31.76	16.45	7.81	7.22	386.46	1.39	0.1	13.84	32.32	128.3	12.83	2.04
TL-S08	1.42	46.53	25.99	53.48	51.32	26.06	22.62	7.06	6.48	202.34	1.39	0.13	10.77	32.59	197.64	11.47	2.7
TL-S09	1.21	54.32	26.91	39	35.52	29.21	35.27	6.27	5.5	220.82	2.37	0.23	10.29	32.45	153.47	7.69	2.58
TL-S10	1.47	44.63	20.32	32.96	48.81	32.29	18.9	7.89	7.53	395.4	0.81	0.06	13.13	58.43	178.25	10.33	2.04
TL-S11	1.34	49.46	10.42	35.86	46.05	29.96	23.99	8.14	7.74	428.46	1.15	0.14	7.97	15.4	167.81	11.47	2.15
TL-S12	1.4	47.11	12.69	38.02	54.79	40.26	5	7.73	7.14	559.91	0.77	0.1	7.62	26.99	174.07	14.68	4.76
TL-S13	1.48	44.09	18.82	27.63	43.46	12.82	43.72	7.63	6.96	415.51	0.71	0.08	8.69	18.91	103.98	20.39	2.1
TL-S14	1.48	43.99	20.91	26.08	47.55	36.22	16.23	8.17	7.53	798.61	0.83	0.09	9.35	36.8	180.45	12.33	3.33
TL-S15	1.38	48.01	15.27	31.42	59.85	35.93	5	7.83	7.04	492.21	1.32	0.1	13.21	45.76	91.49	16.78	3.32
TL-S16	1.44	45.67	12.99	32.92	64.71	10	25.82	7.71	7.24	481.65	1.32	0.09	14.48	43.94	247.73	9.4	3.6
TL-S17	1.4	47.3	24.93	43.06	51.51	42.46	6.02	7.09	6.48	235.77	1.67	0.16	10.48	43.9	107.87	13.35	2.69
TL-S18	1.34	49.26	13.31	32.89	59.54	35.5	5	7.25	6.69	465.65	2.09	0.07	20	37.12	201.21	12.83	1.43
TL-S19	1.3	50.84	20.36	26.51	59.81	34.21	5.98	7.11	6.74	353.11	2.45	0.06	20	36.34	165.81	8.41	2.71
TL-S20	1.32	50.24	18.28	37.07	46.62	40.07	13.32	6.68	6.03	309.99	1.87	0.15	12.07	43.62	132.82	3.99	3.56
TL-S21	1.07	59.55	18.03	44.64	49.19	36.66	14.15	6.62	6.31	328.57	1.6	0.11	14.54	30.62	144.84	9.52	2.74
TL-S22	1.17	55.91	24.61	40.4	44.06	39.98	15.96	6.46	5.74	239.22	2.5	0.19	12.96	31	130.03	8.4	3.25

https://doi.org/10.3390/ijms27041856

TL-S23	1.15	56.51	14.6	36.67	42.14	39.82	18.04	6.59	6.19	331.22	2.45	0.13	19.34	23.95	75.92	9.86	2.24
TL-S24	1.48	44.04	15.45	39.92	46.57	18.19	35.24	7.59	7.15	371.49	1.47	0.09	15.6	44.19	257.8	20.23	3.05
TL-S25	1.48	44.1	10.67	22.46	29.27	21.06	49.67	8.02	7.56	348.52	1.43	0.1	14.44	44.17	151.68	13.15	3.13
TL-S26	1.41	46.87	23.12	50.6	45.73	38.48	15.78	6.53	6.07	313.83	1.83	0.16	11.41	30.32	177.99	8.73	2.43
TL-S27	1.25	52.8	8.02	40.18	58.95	24.24	16.8	7.74	7.12	562.27	0.57	0.09	6.31	33.29	190.93	9.32	2.79
TL-S28	1.38	47.88	17.59	41.77	40.3	22.9	36.8	7.78	7.39	544.9	1.44	0.12	11.98	41.58	181.62	18.69	2.75
TL-S29	1.4	47.32	18.59	20	42.13	16.51	41.36	7.71	6.99	504.28	0.67	0.12	6	42.44	124.62	16.12	3.46
TL-S30	1.42	46.44	10.99	25.81	37.56	22.89	39.56	7.91	7.46	485.57	2.11	0.1	20	48.87	162.82	12.04	3.38
TL-S31	1.47	44.57	8.18	40.02	62.48	27.98	9.54	7.51	6.88	441.84	1.4	0.07	19.24	48.57	259.88	10.47	2.41
TL-S32	1.28	51.52	8.88	29.46	44.79	36.5	18.71	8.12	7.8	303.32	1.4	0.15	9.05	32.47	141.35	15.62	3.53
TL-S33	1.31	50.66	15.79	27.16	37.17	35.71	27.12	7.46	6.72	514.56	0.89	0.11	8.28	43.23	142.12	15.41	2.63

Notes: Soil samples (TL-S01–TL-S33) were collected beneath Tilia spp. in the Oradea region (June 2025). BD (g cm^{-3}) is bulk density determined by the core method; TP (%) is total porosity computed from BD assuming particle density = 2.65 g cm^{-3} $TP = 100 \times (1 - BD/2.65)$, reflecting soil aeration/compaction. GM (%) is gravimetric moisture (105 °C oven-dry), and WHC (%) is water-holding capacity at near-field saturation, indicating short-term water availability. Sand/Silt/Clay (%) are particle-size fractions (pipette/hydrometer per ISO 11277), summing ~100% and defining texture-driven hydraulic behavior. pH(H_2O) and pH(KCl) were measured potentiometrically in 1:2.5 soil–solution suspensions; pH(KCl) is typically ~0.3–0.8 units lower and reflects exchangeable acidity. EC (μS cm^{-1}) is electrical conductivity (1:5 soil–water extract, ISO 11265), an index of soluble salts/salinity. Corg (%) (Walkley–Black, ISO 14235) and Nt (%) (Kjeldahl, ISO 11261) describe organic matter and total N pools; C/N (–) is calculated as Corg/Nt (both in %), indicating organic matter stability and mineralization potential. Pavail (mg kg^{-1}) is plant-available P extracted by Olsen/Egner-Riehm/Mehlich (method selected by pH); Kex (mg kg^{-1}), Caex and Mgex (cmol(+) kg^{-1}) are exchangeable cations (ammonium-acetate extraction; quantified by flame photometry/AAS or titrimetry), representing base status and nutrient supply. Numerics are reported with consistent units and rounding; texture classes, pH, and EC together frame fertility and potential anthropogenic influence (urban sites generally show higher pH and EC; forested sites tend to have higher Corg and more buffered C/N). These variables jointly characterize edaphic conditions governing nutrient dynamics, water regime, and Tilia performance across urban, local, and forest habitats. Abbreviations: BD = bulk density; TP = total porosity; GM = gravimetric moisture; WHC = water-holding capacity; EC = electrical conductivity; Corg = organic carbon; Nt = total nitrogen; Pavail = available phosphorus; Kex/Caex/Mgex = exchangeable K/Ca/Mg.

Table S1b. Physical and Chemical Properties of Soil Samples Corresponding to Tilia Trees

Identification Code	MR (mg CO_2/kg/h)	CAT (mL O_2/g/min)	DHG (µg TPF/g/h)	PHO (µg PNP/g/h)	HUM (%)
TL-S01	1.36	3.33	45.78	162.64	5.1
TL-S02	1.12	2.02	39.13	97.81	1.89
TL-S03	1.07	2.14	22.54	118.67	2.58
TL-S04	0.9	1.57	28.86	104.26	1.43
TL-S05	1.15	1.57	22.1	124.42	2.47
TL-S06	0.72	1.7	14.4	99.83	2.7
TL-S07	1.41	2.22	24.85	153.9	1.49
TL-S08	0.66	2.33	21.5	115.46	2.57
TL-S09	1.41	4.08	29.79	193.54	3.48
TL-S10	1.39	1.91	18.71	143.19	2.46
TL-S11	0.95	1.97	33.26	112.72	2.59
TL-S12	0.95	1.45	20.21	117.27	2.31
TL-S13	0.95	1.27	17.76	83.69	2.14
TL-S14	1.13	1.61	20.93	68.75	2.06
TL-S15	1.12	1.42	13.1	145.73	2.36
TL-S16	0.93	1.89	24.37	98.84	1.15
TL-S17	1.43	2.61	25.63	132.33	2.51
TL-S18	1.09	1.69	28.26	87.42	2.53
TL-S19	1.55	1.91	34.51	167.4	3.16
TL-S20	1.7	3.6	46.57	193.67	4.64
TL-S21	1.13	1.41	38.54	141.13	4.57
TL-S22	0.9	2.02	23.18	66.66	3.66
TL-S23	2.39	1.97	41.93	118.38	4.58
TL-S24	1.32	1.63	30.92	121.5	2.12
TL-S25	0.86	1.65	37.5	143.18	1.62
TL-S26	1.68	2.7	30.21	177.21	3.52

TL-S27	0.55	0.5	17.65	182.61	2.46
TL-S28	1.4	1.7	33.23	96.72	3.07
TL-S29	0.75	1.92	20.57	102.4	2.1
TL-S30	0.98	1.97	22.41	87.03	2.74
TL-S31	1.11	0.6	12.75	101.19	1.56
TL-S32	1.07	0.91	26.12	92.67	2.96
TL-S33	1.07	1.49	33.61	96.78	2.22

Notes: Soil biological and biochemical indicators were analyzed to assess microbial activity and organic matter dynamics in samples (TL-S01–TL-S33) collected beneath *Tilia* spp. in the Oradea region (June 2025). MR (mg CO_2 kg^{-1} h^{-1}) represents microbial respiration, determined by CO_2 evolution from incubated soil, indicating total microbial metabolic activity and soil aeration. CAT (mL O_2 g^{-1} min^{-1}) denotes catalase activity, measured volumetrically via oxygen release from H_2O_2 decomposition, reflecting oxidative enzyme potential and biological stress response. DHG (µg TPF g^{-1} h^{-1}) refers to dehydrogenase activity, assessed by the reduction of triphenyltetrazolium chloride (TTC) to triphenyl formazan (TPF), a proxy for overall microbial redox metabolism and active biomass. PHO (µg PNP g^{-1} h^{-1}) represents phosphatase activity, quantified colorimetrically from p-nitrophenol (PNP) release, and reflects the mineralization rate of organic phosphorus and nutrient cycling intensity. HUM (%) denotes the humus content derived from organic carbon conversion (Corg × 1.724), describing the long-term pool of stabilized organic matter. Collectively, these biochemical indices provide an integrated assessment of soil biological quality, fertility, and ecosystem functioning. Higher MR, DHG, and PHO values are typically associated with elevated microbial activity and nutrient turnover in forest soils, while lower enzyme activities in urban samples may indicate compaction, pollutant stress, or reduced organic inputs. *Abbreviations:* MR = microbial respiration; CAT = catalase activity; DHG = dehydrogenase activity; PHO = phosphatase activity; HUM = humus content.

Table S2. Concentrations of Priority Heavy Metals in Soil Samples Collected Beneath *Tilia* Species in the Oradea Region

Soil ID	Species	Habitat	Pb (mg/kg)	Cd (mg/kg)	Zn (mg/kg)	Cu (mg/kg)	Ni (mg/kg)	Cr (mg/kg)	Mn (mg/kg)	Co (mg/kg)	As (mg/kg)	Hg (mg/kg)	Al (mg/kg)	V (mg/kg)
TL-S01	*T. tomentosa*	Forest soil	22.37 ± 0.89	0.14 ± 0.03	42.6 ± 3.1	10.8 ± 0.9	12.7 ± 1.0	15.8 ± 1.2	412.5 ± 28.7	9.8 ± 0.5	0.21 ± 0.03	0.006 ± 0.001	1487.36 ± 54.12	34.6 ± 1.8
TL-S02	*T. cordata*	Urban soil	35.17 ± 2.18	0.26 ± 0.05	68.4 ± 4.7	17.6 ± 1.3	18.9 ± 1.4	24.7 ± 1.5	538.2 ± 32.1	13.2 ± 0.7	0.38 ± 0.04	0.014 ± 0.002	2214.58 ± 73.44	48.3 ± 2.4
TL-S03	*T. tomentosa*	Urban soil	29.23 ± 0.92	0.24 ± 0.04	64.9 ± 4.2	16.9 ± 1.2	17.6 ± 1.3	23.4 ± 1.4	521.7 ± 30.4	12.6 ± 0.7	0.35 ± 0.04	0.012 ± 0.002	2146.73 ± 69.31	45.7 ± 2.3
TL-S04	*T. tomentosa*	Urban soil	27.63 ± 0.83	0.22 ± 0.04	59.7 ± 3.9	15.8 ± 1.1	16.8 ± 1.2	22.1 ± 1.3	507.3 ± 29.6	11.9 ± 0.6	0.33 ± 0.03	0.011 ± 0.002	2083.29 ± 63.18	43.6 ± 2.1
TL-S05	*T. tomentosa*	Urban soil	28.23 ± 0.99	0.25 ± 0.05	66.8 ± 4.4	17.2 ± 1.3	18.1 ± 1.3	24.1 ± 1.5	545.9 ± 33.2	12.8 ± 0.7	0.36 ± 0.04	0.013 ± 0.002	2197.84 ± 71.22	47.1 ± 2.3
TL-S06	*T. cordata*	Urban soil	26.50 ± 0.77	0.21 ± 0.04	58.3 ± 4.1	15.3 ± 1.1	16.2 ± 1.1	21.6 ± 1.3	496.8 ± 28.9	11.6 ± 0.6	0.32 ± 0.03	0.010 ± 0.002	2039.47 ± 59.36	42.8 ± 2.1
TL-S07	*T. tomentosa*	Urban soil	29.57 ± 0.61	0.27 ± 0.05	71.2 ± 5.0	18.1 ± 1.4	19.3 ± 1.4	25.8 ± 1.6	562.4 ± 34.5	13.1 ± 0.7	0.40 ± 0.04	0.015 ± 0.002	2326.91 ± 78.25	49.6 ± 2.5
TL-S08	*T. platyphyllos*	Local soil	23.17 ± 0.81	0.16 ± 0.03	52.4 ± 3.6	12.3 ± 1.0	14.1 ± 1.0	18.6 ± 1.2	454.1 ± 27.8	10.4 ± 0.5	0.25 ± 0.03	0.007 ± 0.001	1724.63 ± 58.77	36.1 ± 1.9
TL-S09	*T. cordata*	Forest soil	20.03 ± 1.06	0.13 ± 0.03	39.8 ± 2.9	9.6 ± 0.8	11.7 ± 0.9	14.1 ± 1.1	397.6 ± 25.9	8.7 ± 0.5	0.18 ± 0.02	0.005 ± 0.001	1386.54 ± 49.13	31.4 ± 1.7
TL-S10	*T. tomentosa*	Urban soil	28.37 ± 1.11	0.23 ± 0.04	63.7 ± 4.6	16.5 ± 1.2	17.3 ± 1.3	23.0 ± 1.4	529.3 ± 31.7	12.3 ± 0.6	0.34 ± 0.03	0.012 ± 0.002	2127.49 ± 66.82	44.9 ± 2.2
TL-S11	*T. cordata*	Urban soil	27.00 ± 0.90	0.22 ± 0.04	57.6 ± 3.8	15.7 ± 1.1	16.4 ± 1.1	21.9 ± 1.3	505.2 ± 29.8	11.5 ± 0.6	0.31 ± 0.03	0.011 ± 0.002	1998.72 ± 61.40	42.0 ± 2.0
TL-S12	*T. platyphyllos*	Urban soil	27.03 ± 1.18	0.24 ± 0.05	62.1 ± 4.3	16.2 ± 1.2	17.0 ± 1.2	22.8 ± 1.4	518.7 ± 30.6	12.0 ± 0.6	0.32 ± 0.03	0.011 ± 0.002	2072.58 ± 64.15	44.1 ± 2.2
TL-S13	*T. cordata*	Urban soil	28.33 ± 0.76	0.23 ± 0.04	60.5 ± 4.0	15.9 ± 1.1	16.7 ± 1.2	22.3 ± 1.4	512.9 ± 30.1	11.8 ± 0.6	0.33 ± 0.03	0.012 ± 0.002	2034.19 ± 60.52	43.3 ± 2.1
TL-S14	*T. cordata*	Urban soil	27.07 ± 0.70	0.21 ± 0.04	58.9 ± 3.7	15.4 ± 1.1	16.3 ± 1.1	21.7 ± 1.3	501.6 ± 29.3	11.2 ± 0.5	0.30 ± 0.03	0.010 ± 0.002	1979.83 ± 57.29	41.8 ± 2.0
TL-S15	*T. tomentosa*	Urban soil	28.90 ± 0.80	0.25 ± 0.05	67.3 ± 4.8	17.8 ± 1.3	18.4 ± 1.3	24.4 ± 1.5	549.8 ± 33.6	12.9 ± 0.7	0.37 ± 0.04	0.014 ± 0.002	2241.67 ± 75.60	47.8 ± 2.3
TL-S16	*T. tomentosa*	Urban soil	28.53 ± 0.75	0.24 ± 0.04	61.7 ± 4.2	16.7 ± 1.2	17.2 ± 1.2	23.2 ± 1.4	527.1 ± 31.4	12.2 ± 0.6	0.34 ± 0.03	0.012 ± 0.002	2108.21 ± 67.14	44.6 ± 2.2
TL-S17	*T. tomentosa*	Local soil	23.87 ± 0.85	0.17 ± 0.03	54.1 ± 3.5	13.1 ± 1.1	14.7 ± 1.1	19.1 ± 1.3	462.3 ± 28.1	10.7 ± 0.5	0.24 ± 0.03	0.007 ± 0.001	1819.44 ± 53.92	37.2 ± 1.9
TL-S18	*T. cordata*	Local soil	23.57 ± 0.75	0.16 ± 0.03	51.8 ± 3.4	12.7 ± 1.0	14.2 ± 1.0	18.4 ± 1.2	451.7 ± 27.4	10.2 ± 0.5	0.23 ± 0.03	0.006 ± 0.001	1764.31 ± 51.86	36.5 ± 1.9
TL-S19	*T. tomentosa*	Local soil	22.60 ± 0.78	0.15 ± 0.03	49.7 ± 3.2	11.9 ± 0.9	13.6 ± 1.0	17.6 ± 1.2	443.8 ± 27.0	9.9 ± 0.5	0.22 ± 0.03	0.006 ± 0.001	1693.27 ± 50.44	35.7 ± 1.8
TL-S20	*T. tomentosa*	Forest soil	19.90 ± 0.80	0.12 ± 0.02	38.4 ± 2.8	9.2 ± 0.7	11.0 ± 0.8	13.7 ± 1.0	384.2 ± 24.9	8.4 ± 0.4	0.16 ± 0.02	0.004 ± 0.001	1357.68 ± 48.39	30.8 ± 1.7
TL-S21	*T. platyphyllos*	Forest soil	19.47 ± 0.80	0.11 ± 0.02	37.9 ± 2.7	8.9 ± 0.7	10.8 ± 0.8	13.3 ± 1.0	378.6 ± 24.3	8.1 ± 0.4	0.15 ± 0.02	0.004 ± 0.001	1316.42 ± 47.81	30.1 ± 1.6
TL-S22	*T. tomentosa*	Forest soil	20.60 ± 0.93	0.13 ± 0.03	40.6 ± 3.1	10.1 ± 0.8	11.4 ± 0.9	14.3 ± 1.1	392.7 ± 25.5	8.9 ± 0.4	0.17 ± 0.02	0.005 ± 0.001	1428.53 ± 52.17	32.5 ± 1.8
TL-S23	*T. tomentosa*	Forest soil	19.63 ± 0.77	0.12 ± 0.02	39.3 ± 2.9	9.4 ± 0.7	11.1 ± 0.9	13.8 ± 1.0	386.4 ± 25.1	8.6 ± 0.4	0.16 ± 0.02	0.005 ± 0.001	1379.28 ± 48.75	31.8 ± 1.7
TL-S24	*T. platyphyllos*	Urban soil	27.43 ± 0.61	0.23 ± 0.04	63.5 ± 4.3	16.4 ± 1.2	17.5 ± 1.3	23.5 ± 1.4	533.5 ± 32.0	12.5 ± 0.6	0.33 ± 0.03	0.012 ± 0.002	2094.36 ± 63.90	45.2 ± 2.2

TL-S25	*T. tomentosa*	Urban soil	26.80 ± 0.92	0.22 ± 0.04	59.1 ± 3.9	15.6 ± 1.1	16.5 ± 1.2	22.0 ± 1.3	514.8 ± 31.0	11.9 ± 0.6	0.31 ± 0.03	0.011 ± 0.002	1983.57 ± 59.21	42.9 ± 2.1
TL-S26	*T. tomentosa*	Forest soil	19.53 ± 1.06	0.11 ± 0.02	38.2 ± 3.0	9.0 ± 0.8	10.7 ± 0.9	13.1 ± 1.0	381.9 ± 24.7	8.2 ± 0.4	0.15 ± 0.02	0.004 ± 0.001	1348.91 ± 49.82	29.8 ± 1.6
TL-S27	*T. tomentosa*	Urban soil	27.83 ± 0.60	0.24 ± 0.04	64.8 ± 4.4	16.8 ± 1.2	17.6 ± 1.3	23.1 ± 1.4	526.9 ± 31.5	12.3 ± 0.6	0.34 ± 0.03	0.013 ± 0.002	2087.72 ± 61.47	45.0 ± 2.2
TL-S28	*T. tomentosa*	Urban soil	29.13 ± 0.85	0.26 ± 0.05	69.7 ± 4.9	17.9 ± 1.3	18.7 ± 1.4	25.1 ± 1.5	553.7 ± 33.9	13.0 ± 0.7	0.36 ± 0.04	0.014 ± 0.002	2294.18 ± 77.33	48.9 ± 2.4
TL-S29	*T. tomentosa*	Urban soil	27.63 ± 0.96	0.23 ± 0.04	61.4 ± 4.0	16.1 ± 1.2	16.9 ± 1.2	22.4 ± 1.3	518.2 ± 31.2	12.1 ± 0.6	0.32 ± 0.03	0.011 ± 0.002	2046.95 ± 62.04	44.0 ± 2.2
TL-S30	*T. tomentosa*	Urban soil	28.13 ± 0.75	0.24 ± 0.04	62.9 ± 4.3	16.3 ± 1.2	17.1 ± 1.2	23.0 ± 1.4	524.6 ± 31.8	12.3 ± 0.6	0.33 ± 0.03	0.012 ± 0.002	2113.84 ± 65.29	44.7 ± 2.2
TL-S31	*T. platyphyllos*	Urban soil	26.57 ± 0.62	0.21 ± 0.04	58.4 ± 3.8	15.2 ± 1.1	16.0 ± 1.1	21.8 ± 1.3	503.7 ± 30.2	11.4 ± 0.6	0.29 ± 0.03	0.010 ± 0.002	1961.73 ± 58.18	41.5 ± 2.0
TL-S32	*T. platyphyllos*	Urban soil	27.87 ± 0.75	0.23 ± 0.04	63.1 ± 4.2	16.6 ± 1.2	17.3 ± 1.2	23.4 ± 1.4	531.1 ± 32.3	12.4 ± 0.6	0.34 ± 0.03	0.013 ± 0.002	2076.42 ± 64.51	46.1 ± 2.3
TL-S33	*T. cordata*	Urban soil	27.20 ± 0.80	0.22 ± 0.04	60.7 ± 4.1	15.8 ± 1.1	16.6 ± 1.2	22.5 ± 1.3	510.4 ± 30.7	11.6 ± 0.6	0.30 ± 0.03	0.011 ± 0.002	2014.66 ± 60.93	42.3 ± 2.1
	Mean		25.91	3.72	14.35	19.47	35.17	25.91	3.72	14.35	19.47	35.17	25.91	3.72
	SD		0.2015	0.0501	24.88	0.11	0.27	0.2015	0.0501	24.88	0.11	0.27	0.2015	0.0501
	RSD%		56.65	10.23	18.05	37.9	71.2	56.65	10.23	18.05	37.9	71.2	56.65	10.23
	CV (%)		14.51	3.00	20.69	8.9	18.1	14.51	3.00	20.69	8.9	18.1	14.51	3.00
	Min.		15.66	2.63	16.80	10.8	19.3	15.66	2.63	16.80	10.8	19.3	15.66	2.63
	Max.		20.78	3.72	17.91	13.1	25.8	20.78	3.72	17.91	13.1	25.8	20.78	3.72
	Urban soil		27.68	0.23	62.41	16.39	17.33	22.90	516.09	12.04	0.33	0.0119	2079.95	44.39
	Forest soil		20.02	0.13	40.87	9.72	11.72	14.43	396.94	8.68	0.18	0.0049	1427.88	32.38
	T. tomentosa		26.55	0.22	59.87	15.68	16.45	22.25	507.58	11.81	0.32	0.0110	2090.14	44.01
	T. cordata		27.07	0.20	57.67	15.63	16.71	21.58	505.95	11.71	0.30	0.0109	2034.55	43.21
	T. platyphyllos		26.52	0.20	59.16	14.56	15.66	22.08	490.01	11.59	0.28	0.0105	1955.99	41.97

Data represent mean concentrations (± SD) obtained for each metal across all sampled locations. Soil categories reflect contrasting environmental pressures: urban sites are influenced predominantly by traffic emissions and industrial particulate deposition, forest sites correspond to minimally impacted reference areas, and local soils indicate moderate land-use intensity. Variation among Tilia species (*T. tomentosa, T. cordata, and T. platyphyllos*) highlights species-specific differences in rhizosphere interactions and metal accumulation dynamics. The dataset provides a high-resolution geochemical perspective on spatial heterogeneity in metal distributions and supports the suitability of Tilia as an ecological biomonitor for assessing urban–forest gradients in the Oradea region.

Table S3. Concentrations of Priority Heavy Metals in Bark Samples Collected from Tilia Trees in the Oradea Region

Bark ID	Species	Habitat	Pb (mg/kg)	Cd (mg/kg)	Zn (mg/kg)	Cu (mg/kg)	Ni (mg/kg)	Cr (mg/kg)	Mn (mg/kg)	Co (mg/kg)	As (mg/kg)	Hg (mg/kg)	Al (mg/kg)	V (mg/kg)
TL-B01	*T. tomentosa*	Forest soil	3.10 ± 0.16	0.038 ± 0.004	11.8 ± 0.97	2.90 ± 0.21	2.40 ± 0.18	1.60 ± 0.11	46.5 ± 2.79	1.05 ± 0.084	0.0016 ± 0.0002	BLD	263.78 ± 9.23	6.10 ± 0.24
TL-B02	*T. cordata*	Urban soil	9.20 ± 0.46	0.095 ± 0.008	24.6 ± 1.73	4.90 ± 0.30	5.10 ± 0.31	3.10 ± 0.20	82.5 ± 4.95	1.95 ± 0.156	BLD	BLD	413.06 ± 14.46	10.06 ± 0.40
TL-B03	*T. tomentosa*	Urban soil	8.70 ± 0.43	0.089 ± 0.008	23.4 ± 1.64	5.10 ± 0.31	5.35 ± 0.32	3.25 ± 0.21	84.7 ± 5.08	2.05 ± 0.164	BLD	BLD	389.44 ± 13.63	12.88 ± 0.52
TL-B04	*T. tomentosa*	Urban soil	9.80 ± 0.49	0.093 ± 0.009	25.1 ± 1.82	5.30 ± 0.32	5.60 ± 0.34	3.40 ± 0.22	86.9 ± 5.21	2.15 ± 0.172	BLD	BLD	378.12 ± 13.23	11.60 ± 0.46
TL-B05	*T. tomentosa*	Urban soil	10.30 ± 0.52	0.101 ± 0.009	26.3 ± 1.99	5.60 ± 0.34	5.90 ± 0.36	3.65 ± 0.23	89.1 ± 5.35	2.25 ± 0.180	BLD	BLD	395.79 ± 13.85	13.30 ± 0.53
TL-B06	*T. cordata*	Urban soil	8.90 ± 0.45	0.088 ± 0.008	22.9 ± 1.64	5.00 ± 0.30	5.20 ± 0.32	3.15 ± 0.21	91.3 ± 5.48	2.35 ± 0.188	BLD	BLD	388.35 ± 13.59	12.67 ± 0.51
TL-B07	*T. tomentosa*	Urban soil	10.80 ± 0.54	0.106 ± 0.010	27.5 ± 2.05	5.80 ± 0.35	5.95 ± 0.37	3.80 ± 0.24	93.5 ± 5.61	2.45 ± 0.196	0.0032 ± 0.0003	BLD	414.87 ± 14.52	10.00 ± 0.40
TL-B08	*T. platyphyllos*	Local soil	5.20 ± 0.26	0.061 ± 0.006	17.2 ± 1.20	3.90 ± 0.27	3.40 ± 0.24	2.10 ± 0.15	62.4 ± 3.74	1.55 ± 0.124	0.0026 ± 0.0003	BLD	292.13 ± 10.22	7.99 ± 0.32
TL-B09	*T. cordata*	Forest soil	3.40 ± 0.17	0.041 ± 0.004	10.9 ± 0.84	2.80 ± 0.20	2.20 ± 0.16	1.50 ± 0.11	48.2 ± 2.89	1.12 ± 0.090	BLD	BLD	249.06 ± 8.72	6.74 ± 0.27
TL-B10	*T. tomentosa*	Urban soil	9.60 ± 0.48	0.097 ± 0.009	24.1 ± 1.71	5.20 ± 0.32	5.30 ± 0.33	3.30 ± 0.21	95.7 ± 5.74	2.55 ± 0.204	BLD	BLD	400.84 ± 14.03	10.98 ± 0.44
TL-B11	*T. cordata*	Urban soil	9.10 ± 0.46	0.090 ± 0.008	23.0 ± 1.69	4.95 ± 0.30	5.00 ± 0.31	2.95 ± 0.19	97.9 ± 5.87	2.05 ± 0.164	BLD	BLD	383.40 ± 13.42	11.62 ± 0.46
TL-B12	*T. platyphyllos*	Urban soil	10.10 ± 0.51	0.102 ± 0.010	25.8 ± 1.89	5.70 ± 0.34	5.75 ± 0.36	3.70 ± 0.23	100.1 ± 6.01	2.18 ± 0.172	BLD	BLD	397.77 ± 13.92	13.87 ± 0.55
TL-B13	*T. cordata*	Urban soil	9.40 ± 0.47	0.094 ± 0.008	24.0 ± 1.76	5.30 ± 0.32	5.40 ± 0.33	3.25 ± 0.21	112.3 ± 6.14	2.14 ± 0.180	BLD	BLD	403.32 ± 14.12	11.90 ± 0.48
TL-B14	*T. cordata*	Urban soil	8.80 ± 0.44	0.087 ± 0.008	22.5 ± 1.64	4.90 ± 0.30	4.90 ± 0.30	2.90 ± 0.19	100.5 ± 6.27	2.33 ± 0.188	BLD	BLD	412.45 ± 14.44	12.93 ± 0.52
TL-B15	*T. tomentosa*	Urban soil	10.50 ± 0.53	0.108 ± 0.010	26.9 ± 1.90	5.90 ± 0.36	6.00 ± 0.38	3.90 ± 0.25	106.7 ± 6.40	2.45 ± 0.196	BLD	BLD	411.36 ± 14.40	11.66 ± 0.47
TL-B16	*T. tomentosa*	Urban soil	9.90 ± 0.50	0.099 ± 0.009	25.0 ± 1.81	5.50 ± 0.33	5.60 ± 0.34	3.50 ± 0.22	88.0 ± 5.28	2.50 ± 0.204	0.0031 ± 0.0003	BLD	402.60 ± 14.09	10.64 ± 0.43
TL-B17	*T. tomentosa*	Local soil	5.50 ± 0.28	0.067 ± 0.006	18.3 ± 1.36	4.10 ± 0.28	3.70 ± 0.26	2.30 ± 0.16	65.1 ± 3.91	1.72 ± 0.138	BLD	BLD	309.33 ± 10.83	8.75 ± 0.35
TL-B18	*T. cordata*	Local soil	5.10 ± 0.26	0.058 ± 0.006	16.8 ± 1.28	3.80 ± 0.26	3.30 ± 0.24	2.00 ± 0.14	67.8 ± 4.07	1.64 ± 0.131	0.0030 ± 0.0001	BLD	303.30 ± 10.62	7.22 ± 0.29
TL-B19	*T. tomentosa*	Local soil	5.30 ± 0.27	0.064 ± 0.006	17.6 ± 1.34	4.00 ± 0.27	3.50 ± 0.25	2.20 ± 0.15	70.2 ± 4.21	1.81 ± 0.145	BLD	BLD	292.38 ± 10.23	8.14 ± 0.33

TL-B20	*T. tomentosa*	Forest soil	3.00 ± 0.15	0.036 ± 0.004	10.4 ± 0.87	2.70 ± 0.19	2.10 ± 0.15	1.45 ± 0.10	49.8 ± 2.99	1.20 ± 0.096	BLD	BLD	234.03 ± 8.19	5.22 ± 0.21
TL-B21	*T. platyphyllos*	Forest soil	3.30 ± 0.17	0.044 ± 0.004	9.8 ± 0.71	2.60 ± 0.18	2.30 ± 0.16	1.30 ± 0.10	51.0 ± 3.06	1.28 ± 0.102	BLD	BLD	260.34 ± 9.11	5.57 ± 0.22
TL-B22	*T. tomentosa*	Forest soil	3.20 ± 0.16	0.039 ± 0.004	11.1 ± 0.83	3.00 ± 0.21	2.60 ± 0.18	1.75 ± 0.12	52.3 ± 3.14	1.35 ± 0.108	BLD	BLD	252.57 ± 8.84	5.25 ± 0.21
TL-B23	*T. tomentosa*	Forest soil	3.50 ± 0.18	0.042 ± 0.004	12.0 ± 0.91	3.10 ± 0.22	2.80 ± 0.20	1.85 ± 0.13	54.1 ± 3.25	1.18 ± 0.094	BLD	BLD	253.54 ± 8.87	6.25 ± 0.25
TL-B24	*T. platyphyllos*	Urban soil	10.20 ± 0.51	0.099 ± 0.009	25.3 ± 1.89	5.40 ± 0.33	5.45 ± 0.34	3.60 ± 0.23	90.2 ± 5.41	2.65 ± 0.212	0.0017 ± 0.0002	BLD	427.66 ± 14.97	11.91 ± 0.48
TL-B25	*T. platyphyllos*	Urban soil	9.70 ± 0.48	0.091 ± 0.008	23.8 ± 1.72	5.10 ± 0.31	5.10 ± 0.31	3.20 ± 0.21	92.4 ± 5.54	2.30 ± 0.184	BLD	BLD	383.01 ± 13.41	10.16 ± 0.41
TL-B26	*T. tomentosa*	Forest soil	3.10 ± 0.16	0.038 ± 0.004	10.7 ± 0.83	2.80 ± 0.20	2.30 ± 0.16	1.55 ± 0.11	55.6 ± 3.34	1.22 ± 0.098	BLD	BLD	244.10 ± 8.54	5.47 ± 0.22
TL-B27	*T. tomentosa*	Urban soil	10.90 ± 0.55	0.106 ± 0.010	27.2 ± 2.04	5.80 ± 0.35	5.85 ± 0.37	3.75 ± 0.24	94.6 ± 5.68	2.10 ± 0.168	BLD	BLD	404.58 ± 14.16	11.65 ± 0.47
TL-B28	*T. tomentosa*	Urban soil	11.20 ± 0.56	0.110 ± 0.010	28.4 ± 2.06	6.00 ± 0.36	6.10 ± 0.39	3.95 ± 0.25	96.8 ± 5.81	2.20 ± 0.176	0.0020 ± 0.0002	BLD	389.38 ± 13.63	13.51 ± 0.54
TL-B29	*T. tomentosa*	Urban soil	10.40 ± 0.52	0.103 ± 0.009	26.0 ± 1.93	5.60 ± 0.34	5.75 ± 0.36	3.60 ± 0.23	99.0 ± 5.94	2.40 ± 0.192	BLD	BLD	397.26 ± 13.90	10.48 ± 0.42
TL-B30	*T. tomentosa*	Urban soil	9.30 ± 0.47	0.090 ± 0.008	23.5 ± 1.69	5.15 ± 0.31	5.15 ± 0.31	3.10 ± 0.20	101.2 ± 6.07	2.50 ± 0.200	BLD	BLD	410.91 ± 14.38	11.25 ± 0.45
TL-B31	*T. tomentosa*	Urban soil	8.60 ± 0.43	0.085 ± 0.008	22.1 ± 1.51	4.85 ± 0.30	4.80 ± 0.29	2.85 ± 0.19	103.4 ± 6.20	2.60 ± 0.208	BLD	BLD	391.78 ± 13.71	10.16 ± 0.41
TL-B32	*T. tomentosa*	Urban soil	9.50 ± 0.48	0.092 ± 0.008	24.3 ± 1.78	5.35 ± 0.32	5.35 ± 0.33	3.25 ± 0.21	105.6 ± 6.34	2.32 ± 0.186	0.0017 ± 0.0001	BLD	390.46 ± 13.67	12.32 ± 0.49
TL-B33	*T. tomentosa*	Urban soil	10.00 ± 0.50	0.101 ± 0.009	25.6 ± 1.86	5.90 ± 0.36	5.95 ± 0.37	3.85 ± 0.24	98.3 ± 5.90	2.12 ± 0.170	BLD	BLD	410.81 ± 14.38	11.83 ± 0.47
TL-B34	*T. cordata*	Urban soil	9.00 ± 0.45	0.089 ± 0.008	23.2 ± 1.65	5.40 ± 0.33	5.10 ± 0.31	3.15 ± 0.21	95.0 ± 5.70	2.22 ± 0.178	BLD	BLD	409.23 ± 14.32	12.57 ± 0.50
	Mean		7.87	0.081	21.09	4.69	4.60	2.88	83.20	1.998	0.00236	BLD	357.68	10.08
	SD		2.86	0.0246	5.92	1.10	1.35	0.84	20.12	0.487	0.00069	BLD	64.94	2.70
	RSD%		36.34	30.48	28.06	23.49	29.40	29.07	24.17	24.36	29.28	BLD	18.15	26.80
	CV (%)		36.34	30.48	28.06	23.49	29.40	29.07	24.17	24.36	29.28	BLD	18.15	26.80
	Min.		3.00	0.036	9.8	2.6	2.1	1.3	46.5	1.05	0.0016	BLD	234.03	5.22
	Max.		11.2	0.110	28.4	6.0	6.1	3.95	112.3	2.65	0.0032	BLD	427.66	13.87
	Urban soil		9.72	0.096	24.74	5.37	5.52	3.37	94.01	2.29	0.0012	0.0001	401.52	11.43
	Forest soil		3.25	0.040	11.19	2.88	2.28	1.57	51.33	1.18	0.0002	0.0000	253.07	5.86
	T. tomentosa		8.37	0.083	22.40	4.90	4.85	3.04	85.73	2.12	0.0012	0.0002	372.61	10.94
	T. cordata		7.38	0.080	21.17	4.50	4.53	2.86	86.86	2.06	0.0001	0.0000	363.77	10.74

T. platyphyllos	7.38	0.078	21.53	4.47	4.57	2.77	82.25	2.17	0.0014	0.0000	356.58	10.03

Data represent mean concentrations (± standard deviation, SD) of heavy metals measured in bark samples collected from *Tilia* species across all sampled locations in the Oradea region. "BLD" indicates values below the analytical detection limit. Habitat categories reflect differences in environmental exposure: urban soils are influenced primarily by vehicular traffic emissions and industrial particulate deposition; forest soils represent low-impact background reference areas; and local soils correspond to moderately impacted zones with mixed land-use activity. Variation among species (*T. tomentosa*, *T. cordata*, and *T. platyphyllos*) highlights species-specific differences in bark sorption capacity and metal accumulation behavior, supporting the suitability of *Tilia* spp. as biomonitors of atmospheric metal pollution along the urban–forest gradient in Oradea.

Table S4. Concentrations of Selected Heavy Metals in *Tilia* Leaf Samples from Different Habitats in the Oradea Region

Bark ID	Species	Habitat	Pb (mg/kg)	Cd (mg/kg)	Zn (mg/kg)	Cu (mg/kg)	Ni (mg/kg)	Cr (mg/kg)	Mn (mg/kg)	Co (mg/kg)	As (mg/kg)	Hg (mg/kg)	Al (mg/kg)	V (mg/kg)
TL-L01	*T. tomentosa*	Forest soil	0.16 ± 0.03	BLD	8.7 ± 1.2	0.6 ± 0.08	0.07 ± 0.01	BLD	4.8 ± 0.6	BLD	BLD	BLD	BLD	BLD
TL-L02	*T. cordata*	Urban soil	0.32 ± 0.05	BLD	19.6 ± 2.3	2.4 ± 0.30	0.32 ± 0.04	0.014 ± 0.002	12.6 ± 1.7	0.028 ± 0.004	BLD	BLD	14.8 ± 1.9	0.006 ± 0.001
TL-L03	*T. tomentosa*	Urban soil	0.30 ± 0.05	BLD	18.9 ± 2.2	2.1 ± 0.28	0.29 ± 0.04	0.012 ± 0.002	11.9 ± 1.5	0.022 ± 0.003	BLD	BLD	12.6 ± 1.7	0.005 ± 0.001
TL-L04	*T. tomentosa*	Urban soil	0.28 ± 0.05	BLD	20.3 ± 2.4	2.6 ± 0.32	0.34 ± 0.04	0.016 ± 0.003	13.1 ± 1.8	0.031 ± 0.004	BLD	BLD	15.9 ± 2.1	0.007 ± 0.001
TL-L05	*T. tomentosa*	Urban soil	0.31 ± 0.05	BLD	21.0 ± 2.5	2.9 ± 0.35	0.37 ± 0.05	0.018 ± 0.003	14.2 ± 1.9	0.035 ± 0.005	BLD	BLD	18.4 ± 2.4	0.008 ± 0.001
TL-L06	*T. cordata*	Urban soil	0.33 ± 0.06	BLD	19.2 ± 2.3	2.2 ± 0.27	0.30 ± 0.04	BLD	11.4 ± 1.5	BLD	BLD	BLD	11.7 ± 1.6	BLD
TL-L07	*T. tomentosa*	Urban soil	0.34 ± 0.06	BLD	22.1 ± 2.6	3.1 ± 0.38	0.39 ± 0.05	0.020 ± 0.003	14.6 ± 1.9	0.040 ± 0.005	BLD	BLD	19.6 ± 2.5	0.009 ± 0.002
TL-L08	*T. platyphyllos*	Local soil	0.22 ± 0.04	BLD	13.5 ± 1.8	1.3 ± 0.16	0.16 ± 0.02	BLD	8.6 ± 1.1	BLD	BLD	BLD	8.4 ± 1.1	BLD
TL-L09	*T. cordata*	Forest soil	0.15 ± 0.03	BLD	7.9 ± 1.1	0.5 ± 0.07	0.06 ± 0.01	BLD	4.1 ± 0.5	BLD	BLD	BLD	BLD	BLD
TL-L10	*T. tomentosa*	Urban soil	0.29 ± 0.05	BLD	18.7 ± 2.2	2.0 ± 0.26	0.28 ± 0.04	0.011 ± 0.002	10.8 ± 1.4	0.018 ± 0.003	BLD	BLD	10.9 ± 1.5	0.004 ± 0.001
TL-L11	*T. cordata*	Urban soil	0.27 ± 0.05	BLD	17.5 ± 2.1	1.8 ± 0.24	0.25 ± 0.03	BLD	10.1 ± 1.3	BLD	BLD	BLD	9.6 ± 1.4	BLD
TL-L12	*T. platyphyllos*	Urban soil	0.31 ± 0.05	BLD	21.8 ± 2.6	3.0 ± 0.36	0.38 ± 0.05	0.017 ± 0.003	13.8 ± 1.8	0.033 ± 0.004	BLD	BLD	17.8 ± 2.3	0.007 ± 0.001
TL-L13	*T. cordata*	Urban soil	0.33 ± 0.06	BLD	20.6 ± 2.4	2.5 ± 0.30	0.33 ± 0.04	BLD	12.4 ± 1.6	BLD	BLD	BLD	11.5 ± 1.6	BLD
TL-L14	*T. cordata*	Urban soil	0.28 ± 0.05	BLD	19.9 ± 2.3	2.1 ± 0.27	0.29 ± 0.04	BLD	11.3 ± 1.5	BLD	BLD	BLD	10.2 ± 1.4	BLD
TL-L15	*T. tomentosa*	Urban soil	0.35 ± 0.06	BLD	22.8 ± 2.7	3.2 ± 0.40	0.40 ± 0.05	0.021 ± 0.003	14.9 ± 2.0	0.041 ± 0.005	BLD	BLD	20.1 ± 2.7	0.010 ± 0.002
TL-L16	*T. tomentosa*	Urban soil	0.30 ± 0.05	BLD	18.3 ± 2.2	1.9 ± 0.25	0.26 ± 0.03	BLD	10.5 ± 1.4	BLD	BLD	BLD	9.8 ± 1.3	BLD
TL-L17	*T. tomentosa*	Local soil	0.23 ± 0.04	BLD	14.4 ± 1.9	1.4 ± 0.17	0.18 ± 0.02	BLD	8.1 ± 1.0	BLD	BLD	BLD	7.1 ± 1.0	BLD
TL-L18	*T. cordata*	Local soil	0.21 ± 0.04	BLD	12.8 ± 1.8	1.1 ± 0.14	0.14 ± 0.02	BLD	7.6 ± 0.9	BLD	BLD	BLD	6.4 ± 0.9	BLD
TL-L19	*T. tomentosa*	Local soil	0.24 ± 0.04	BLD	15.1 ± 2.0	1.5 ± 0.18	0.19 ± 0.02	BLD	8.4 ± 1.1	BLD	BLD	BLD	7.9 ± 1.1	BLD
TL-L20	*T. tomentosa*	Forest soil	0.14 ± 0.02	BLD	8.4 ± 1.2	0.7 ± 0.08	0.08 ± 0.01	BLD	4.4 ± 0.6	BLD	BLD	BLD	BLD	BLD
TL-L21	*T. platyphyllos*	Forest soil	0.17 ± 0.03	BLD	10.6 ± 1.5	0.8 ± 0.09	0.10 ± 0.01	BLD	5.3 ± 0.7	BLD	BLD	BLD	BLD	BLD
TL-L22	*T. tomentosa*	Forest soil	0.18 ± 0.03	BLD	11.2 ± 1.5	0.9 ± 0.10	0.11 ± 0.01	BLD	5.9 ± 0.8	BLD	BLD	BLD	BLD	BLD
TL-L23	*T. tomentosa*	Forest soil	0.16 ± 0.03	BLD	9.5 ± 1.3	0.6 ± 0.07	0.09 ± 0.01	BLD	BLD	BLD	BLD	BLD	BLD	BLD
TL-L24	*T. platyphyllos*	Urban soil	0.29 ± 0.05	BLD	21.3 ± 2.6	2.8 ± 0.34	0.36 ± 0.04	0.015 ± 0.002	13.3 ± 1.8	0.029 ± 0.004	BLD	BLD	16.7 ± 2.2	0.006 ± 0.001

TL-L25	*T. platyphyllos*	Urban soil	0.32 ± 0.05	BLD	18.9 ± 2.2	2.3 ± 0.28	0.31 ± 0.04	BLD	11.8 ± 1.5	BLD	BLD	BLD	12.3 ± 1.7	0.005 ± 0.001
TL-L26	*T. tomentosa*	Forest soil	0.15 ± 0.03	BLD	9.1 ± 1.3	0.5 ± 0.06	0.07 ± 0.01	BLD	BLD	BLD	BLD	BLD	BLD	BLD
TL-L27	*T. tomentosa*	Urban soil	0.34 ± 0.06	BLD	22.0 ± 2.6	2.9 ± 0.36	0.37 ± 0.05	0.018 ± 0.003	13.9 ± 1.9	0.038 ± 0.005	BLD	BLD	18.9 ± 2.5	0.007 ± 0.001
TL-L28	*T. tomentosa*	Urban soil	0.30 ± 0.05	BLD	23.4 ± 2.8	3.1 ± 0.39	0.41 ± 0.05	0.022 ± 0.004	14.7 ± 2.0	0.044 ± 0.006	BLD	BLD	21.5 ± 2.8	0.009 ± 0.002
TL-L29	*T. tomentosa*	Urban soil	0.33 ± 0.06	BLD	20.8 ± 2.5	2.4 ± 0.30	0.34 ± 0.04	0.013 ± 0.002	12.9 ± 1.7	0.030 ± 0.004	BLD	BLD	15.6 ± 2.1	0.006 ± 0.001
TL-L30	*T. tomentosa*	Urban soil	0.31 ± 0.05	BLD	19.4 ± 2.3	2.1 ± 0.26	0.30 ± 0.04	BLD	11.6 ± 1.5	BLD	BLD	BLD	10.7 ± 1.4	BLD
TL-L31	*T. tomentosa*	Urban soil	0.29 ± 0.05	BLD	18.0 ± 2.2	1.7 ± 0.22	0.24 ± 0.03	BLD	10.2 ± 1.3	BLD	BLD	BLD	9.2 ± 1.2	BLD
TL-L32	*T. tomentosa*	Urban soil	0.27 ± 0.04	BLD	21.5 ± 2.6	2.6 ± 0.33	0.35 ± 0.04	BLD	13.5 ± 1.8	BLD	BLD	BLD	17.1 ± 2.2	BLD
TL-L33	*T. tomentosa*	Urban soil	0.35 ± 0.06	BLD	22.6 ± 2.7	3.0 ± 0.38	0.39 ± 0.05	0.019 ± 0.003	14.3 ± 1.9	0.039 ± 0.005	BLD	BLD	20.4 ± 2.7	0.008 ± 0.001
TL-L34	*T. cordata*	Urban soil	0.32 ± 0.05	BLD	20.4 ± 2.4	2.3 ± 0.28	0.33 ± 0.04	BLD	12.1 ± 1.6	BLD	BLD	BLD	12.9 ± 1.7	BLD
	Mean		0.2688	0.0000	17.3588	1.9676	0.2603	0.0064	10.2088	0.0126	0.0000	0.0000	10.8235	0.0029
	SD		0.0666	0.0000	4.8562	0.8605	0.1134	0.0085	4.0812	0.0169	0.0000	0.0000	6.8816	0.0036
	RSD%		24.77	0.00	27.98	43.73	43.57	133.16	39.98	134.09	0.00	0.00	63.58	127.15
	CV (%)		24.77	0.00	27.98	43.73	43.57	133.16	39.98	134.09	0.00	0.00	63.58	127.15
	Min.		0.1400	0.0000	7.9000	0.5000	0.0600	0.0000	0.0000	0.0000	0.0000	0.0000	0.0000	0.0000
	Max.		0.3500	0.0000	23.4000	3.2000	0.4100	0.0220	14.9000	0.0440	0.0000	0.0000	21.5000	0.0100
	Urban soil		0.3091	0.0000	19.8152	2.3830	0.3124	0.0101	12.1296	0.0182	0.0000	0.0000	14.7043	0.0042
	Forest soil		0.1588	0.0000	9.6000	0.6571	0.0836	0.0000	3.9875	0.0000	0.0000	0.0000	0.0000	0.0000
	T. tomentosa		0.2676	0.0000	17.4381	1.9905	0.2629	0.0081	10.1286	0.0161	0.0000	0.0000	11.2238	0.0035
	T. cordata		0.2762	0.0000	17.2375	1.8625	0.2525	0.0018	10.2000	0.0035	0.0000	0.0000	9.6375	0.0008
	T. platyphyllos		0.2620	0.0000	17.2200	2.0400	0.2620	0.0064	10.5600	0.0124	0.0000	0.0000	11.0400	0.0036

Data represent mean concentrations (± standard deviation, SD) of heavy metals measured in leaf samples collected from *Tilia* species across urban and forest habitats in the Oradea region. "BLD" indicates values below the analytical detection limit. Habitat categories reflect contrasting environmental exposure conditions: urban sites are influenced predominantly by vehicular traffic emissions, industrial particulate deposition, and resuspension of road dust, whereas forest sites correspond to low-impact background reference areas minimally affected by anthropogenic inputs. Elemental variation between habitats demonstrates clear enrichment in urban environments, where leaves accumulated significantly higher concentrations of Pb, Zn, Cu, Ni, Mn, Al, and V compared to forest sites, confirming the substantial contribution of atmospheric deposition sources in densely trafficked locations. In contrast, forest samples exhibit trace to minimal values for most metals, reflecting natural baseline geochemical conditions. Variation among Tilia species indicates measurable differences in foliar sorption behavior and metal retention efficiency, highlighting the influence of species-specific leaf surface microstructure and physiological characteristics. These results support

the suitability of Tilia spp. leaves as sensitive passive biomonitors for assessing airborne metal contamination and for tracking the spatial distribution of atmospheric pollution across the urban–forest gradient in the Oradea region.

Table S5. Concentrations of Selected Heavy Metals in *Tilia* Flower Samples from Different Habitats in the Oradea Region

Bark ID	Species	Habitat	Pb (mg/kg)	Cd (mg/kg)	Zn (mg/kg)	Cu (mg/kg)	Ni (mg/kg)	Cr (mg/kg)	Mn (mg/kg)	Co (mg/kg)	As (mg/kg)	Hg (mg/kg)	Al (mg/kg)	V (mg/kg)
TL-F01	*T. tomentosa*	Forest	0.16 ± 0.03	BLD	28.4 ± 4.1	11.8 ± 1.9	BLD	BLD	112.4 ± 11.3	BLD	BLD	BLD	42.5 ± 8.2	BLD
TL-F02	*T. cordata*	Urban	0.32 ± 0.05	BLD	74.2 ± 6.5	26.7 ± 2.8	0.88 ± 0.12	BLD	241.8 ± 21.6	BLD	BLD	BLD	182.4 ± 21.6	0.09 ± 0.02
TL-F03	*T. tomentosa*	Urban	0.30 ± 0.05	BLD	78.5 ± 7.1	27.4 ± 2.9	0.93 ± 0.13	0.21 ± 0.04	254.3 ± 22.5	0.05 ± 0.01	BLD	BLD	194.8 ± 22.5	0.11 ± 0.02
TL-F04	*T. tomentosa*	Urban	0.28 ± 0.04	BLD	82.6 ± 7.8	29.6 ± 3.1	1.02 ± 0.14	0.24 ± 0.04	268.7 ± 24.0	0.06 ± 0.01	BLD	BLD	205.6 ± 23.3	0.13 ± 0.02
TL-F05	*T. tomentosa*	Urban	0.31 ± 0.05	BLD	85.3 ± 8.0	30.1 ± 3.2	0.97 ± 0.13	BLD	276.1 ± 24.6	BLD	BLD	BLD	218.3 ± 24.0	BLD
TL-F06	*T. cordata*	Urban	BLD	BLD	89.1 ± 8.6	32.5 ± 3.4	1.05 ± 0.15	0.27 ± 0.05	288.9 ± 25.3	0.07 ± 0.01	BLD	BLD	232.6 ± 25.5	0.15 ± 0.03
TL-F07	*T. tomentosa*	Urban	0.34 ± 0.06	BLD	92.4 ± 9.0	33.8 ± 3.5	1.12 ± 0.16	0.30 ± 0.05	302.4 ± 26.5	0.08 ± 0.01	BLD	BLD	248.1 ± 26.2	0.18 ± 0.03
TL-F08	*T. platyphyllos*	Local	0.22 ± 0.04	BLD	52.6 ± 5.4	18.7 ± 2.2	BLD	BLD	178.6 ± 16.4	BLD	BLD	BLD	96.4 ± 12.8	BLD
TL-F09	*T. cordata*	Forest	0.15 ± 0.03	BLD	25.9 ± 3.8	10.6 ± 1.7	BLD	BLD	96.5 ± 10.8	BLD	BLD	BLD	38.2 ± 7.5	BLD
TL-F10	*T. tomentosa*	Urban	BLD	BLD	81.3 ± 7.6	28.3 ± 3.0	0.95 ± 0.13	0.22 ± 0.04	265.3 ± 23.8	BLD	BLD	BLD	199.3 ± 23.1	0.10 ± 0.02
TL-F11	*T. cordata*	Urban	0.27 ± 0.05	BLD	76.8 ± 6.9	27.1 ± 2.9	0.90 ± 0.12	BLD	251.9 ± 22.9	BLD	BLD	BLD	187.4 ± 21.6	BLD
TL-F12	*T. platyphyllos*	Urban	0.31 ± 0.05	BLD	88.5 ± 8.5	31.4 ± 3.3	1.03 ± 0.14	BLD	280.4 ± 25.0	0.06 ± 0.01	BLD	BLD	215.8 ± 23.9	0.12 ± 0.02
TL-F13	*T. cordata*	Urban	0.33 ± 0.06	BLD	91.2 ± 8.8	33.1 ± 3.4	1.08 ± 0.15	0.26 ± 0.05	297.2 ± 26.0	0.07 ± 0.01	BLD	BLD	229.6 ± 25.1	0.14 ± 0.03
TL-F14	*T. cordata*	Urban	0.28 ± 0.05	BLD	83.7 ± 7.9	29.9 ± 3.2	0.99 ± 0.13	BLD	269.6 ± 24.0	BLD	BLD	BLD	206.7 ± 23.3	BLD
TL-F15	*T. tomentosa*	Urban	BLD	BLD	97.5 ± 9.4	35.2 ± 3.7	1.18 ± 0.17	0.34 ± 0.06	318.1 ± 27.4	0.09 ± 0.01	BLD	BLD	261.4 ± 27.0	0.20 ± 0.03
TL-F16	*T. tomentosa*	Urban	BLD	BLD	86.4 ± 8.2	30.4 ± 3.2	1.01 ± 0.14	0.28 ± 0.05	276.9 ± 24.6	BLD	BLD	BLD	217.0 ± 24.0	0.14 ± 0.03
TL-F17	*T. tomentosa*	Local	BLD	BLD	55.7 ± 5.7	19.6 ± 2.3	BLD	BLD	185.9 ± 16.9	BLD	BLD	BLD	104.3 ± 13.6	BLD
TL-F18	*T. cordata*	Local	BLD	BLD	51.2 ± 5.3	17.4 ± 2.1	BLD	BLD	172.8 ± 15.5	BLD	BLD	BLD	92.7 ± 12.1	BLD
TL-F19	*T. tomentosa*	Local	0.24 ± 0.04	BLD	57.9 ± 6.0	20.1 ± 2.3	BLD	BLD	192.4 ± 17.8	BLD	BLD	BLD	113.8 ± 14.4	BLD
TL-F20	*T. tomentosa*	Forest	0.14 ± 0.02	BLD	29.1 ± 4.2	12.3 ± 1.9	BLD	BLD	108.9 ± 11.8	BLD	BLD	BLD	45.1 ± 8.5	BLD
TL-F21	*T. platyphyllos*	Forest	0.17 ± 0.03	BLD	33.8 ± 4.6	13.5 ± 2.0	BLD	BLD	121.4 ± 12.3	BLD	BLD	BLD	53.6 ± 9.2	BLD
TL-F22	*T. tomentosa*	Forest	0.18 ± 0.03	BLD	31.4 ± 4.4	14.2 ± 2.1	BLD	BLD	139.6 ± 13.5	BLD	BLD	BLD	61.4 ± 9.8	BLD
TL-F23	*T. tomentosa*	Forest	BLD	BLD	28.7 ± 4.1	12.9 ± 2.0	BLD	BLD	118.2 ± 11.7	BLD	BLD	BLD	49.2 ± 8.7	BLD
TL-F24	*T. platyphyllos*	Urban	0.29 ± 0.05	BLD	84.7 ± 8.0	30.8 ± 3.3	1.00 ± 0.14	BLD	272.6 ± 24.2	BLD	BLD	BLD	211.6 ± 23.6	BLD

TL-F25	*T. platyphyllos*	Urban	BLD	BLD	90.5 ± 8.7	32.9 ± 3.6	1.09 ± 0.15	0.25 ± 0.05	298.1 ± 26.1	0.05 ± 0.01	BLD	BLD	239.8 ± 25.8	0.12 ± 0.02
TL-F26	*T. tomentosa*	Urban	0.15 ± 0.03	BLD	79.2 ± 7.1	26.2 ± 2.8	0.86 ± 0.12	BLD	246.2 ± 22.1	BLD	BLD	BLD	174.5 ± 20.2	BLD
TL-F27	*T. tomentosa*	Urban	0.34 ± 0.06	BLD	95.3 ± 9.2	34.1 ± 3.6	1.15 ± 0.16	0.32 ± 0.06	311.9 ± 27.0	0.08 ± 0.01	BLD	BLD	255.1 ± 26.8	0.17 ± 0.03
TL-F28	*T. tomentosa*	Urban	BLD	BLD	87.6 ± 8.3	30.7 ± 3.3	1.02 ± 0.14	0.24 ± 0.04	279.5 ± 24.8	BLD	BLD	BLD	220.4 ± 24.3	0.13 ± 0.02
TL-F29	*T. tomentosa*	Urban	BLD	BLD	93.9 ± 9.0	33.5 ± 3.6	1.10 ± 0.15	BLD	301.1 ± 26.3	BLD	BLD	BLD	242.6 ± 25.8	BLD
TL-F30	*T. tomentosa*	Urban	BLD	BLD	85.8 ± 8.1	31.2 ± 3.4	1.04 ± 0.14	0.21 ± 0.04	286.8 ± 25.2	BLD	BLD	BLD	233.2 ± 25.1	0.11 ± 0.02
TL-F31	*T. tomentosa*	Urban	0.29 ± 0.05	BLD	81.4 ± 7.7	28.9 ± 3.1	0.97 ± 0.13	BLD	263.7 ± 23.7	BLD	BLD	BLD	209.6 ± 23.5	BLD
TL-F32	*T. tomentosa*	Urban	0.27 ± 0.04	BLD	78.1 ± 7.1	27.6 ± 2.9	0.92 ± 0.13	0.19 ± 0.04	249.0 ± 22.6	BLD	BLD	BLD	195.7 ± 22.7	BLD
TL-F33	*T. tomentosa*	Urban	0.35 ± 0.06	BLD	98.6 ± 9.5	36.5 ± 3.8	1.20 ± 0.17	0.35 ± 0.06	322.3 ± 27.8	0.10 ± 0.01	BLD	BLD	268.3 ± 27.6	0.21 ± 0.03
TL-F34	*T. cordata*	Urban	BLD	BLD	88.9 ± 8.4	32.1 ± 3.5	1.06 ± 0.15	0.27 ± 0.05	291.2 ± 25.5	0.07 ± 0.01	BLD	BLD	243.5 ± 25.9	0.15 ± 0.03
	Mean		0.167	0.000	72.53	26.21	0.721	0.116	239.43	0.0229	0.000	0.000	176.19	0.066
	SD		0.137	0.000	23.38	7.85	0.479	0.136	68.83	0.0348	0.000	0.000	74.43	0.075
	RSD%		82.1%	0.0%	32.2%	29.97%	66.37%	117.4%	28.75%	151.6%	0.0%	0.0%	42.24%	113.3%
	CV (%)		82.1%	0.0%	32.2%	29.97%	66.37%	117.4%	28.75%	151.6%	0.0%	0.0%	42.24%	113.3%
	Min.		0.000	0.000	25.90	10.60	0.000	0.000	96.50	0.000	0.000	0.000	38.20	0.000
	Max.		0.350	0.000	98.60	36.50	1.200	0.350	322.30	0.100	0.000	0.000	268.30	0.210
	Urban soil		0.185	86.31	30.83	1.022	0.165	279.75	0.033	220.55	0.094	0.185	86.31	30.83
	Forest soil		0.133	29.55	12.55	0.000	0.000	116.17	0.000	48.33	0.000	0.133	29.55	12.55
	T. tomentosa		0.160	73.10	26.40	0.735	0.138	241.89	0.022	179.06	0.070	0.160	73.10	26.40
	T. cordata		0.169	72.63	26.18	0.745	0.100	238.74	0.026	176.64	0.066	0.169	72.63	26.18
	T. platyphyllos		0.198	70.02	25.46	0.624	0.050	230.22	0.022	163.44	0.048	0.198	70.02	25.46

Data represent mean concentrations (± standard deviation, SD) of heavy metals measured in flower samples collected from *Tilia* species across urban and forest habitats in the Oradea region. The abbreviation "BLD" indicates values below the analytical detection limit. Habitat categories reflect contrasting environmental exposure conditions: urban sites are influenced predominantly by vehicular traffic emissions, industrial particulate deposition, and resuspension of road dust, whereas forest sites correspond to low-impact background reference areas minimally affected by anthropogenic inputs. Elemental variation between habitats demonstrates clear enrichment in urban environments, where flowers accumulated significantly higher concentrations of Pb, Zn, Cu, Ni, Mn, Al, and V compared to forest sites, confirming the substantial contribution of atmospheric deposition sources in densely

trafficked locations. In contrast, forest flower samples exhibit trace to minimal values for most metals, reflecting natural baseline geochemical conditions. Variation among *Tilia* species indicates measurable differences in floral sorption behavior and metal retention efficiency, highlighting the influence of species-specific surface morphology, glandular secretions, and physiological characteristics affecting particulate capture and accumulation. These results support the suitability of *Tilia* spp. flowers used for herbal tea as sensitive passive biomonitors for assessing airborne metal contamination, and emphasize the importance of evaluating harvest location to ensure pharmacological quality and consumer safety when floral material is collected from polluted urban area

Table S6. Concentrations of Heavy Metals in Tilia Flower Infusions Prepared Under Different Brewing Conditions (μg/L)

Sample Code	Water Type	Temp (°C)	Time (min)	Species (Tilia)	Pb (μg/L)	Cd (μg/L)	Zn (μg/L)	Cu (μg/L)	Ni (μg/L)	Cr (μg/L)	Mn (μg/L)	Co (μg/L)	As (μg/L)	Hg (μg/L)	Al (μg/L)	V (μg/L)
INF-T1	Ultra-pure	70	5	*T. tomentosa*	LOD	0.006 ± 0.001	LOD	0.6 ± 0.05	LOD	LOD	4.8 ± 0.4	LOD	LOD	LOD	LOD	0.06 ± 0.01
INF-T2		80	7		LOD	LOD	3.2 ± 0.3	LOD	LOD	0.08 ± 0.01	LOD	0.03 ± 0.01	LOD	LOD	3.5 ± 0.3	LOD
INF-T3		85	10		LOD	LOD	LOD	LOD	LOD	0.12 ± 0.01	6.5 ± 0.5	LOD	LOD	LOD	4.2 ± 0.3	0.10 ± 0.01
INF-T4		90	12		LOD	0.007 ± 0.001	4.5 ± 0.4	0.8 ± 0.06	0.25 ± 0.03	LOD	10.2 ± 0.8	0.05 ± 0.01	LOD	LOD	5.1 ± 0.4	0.14 ± 0.01
INF-T5		95	15		0.12 ± 0.014	0.010 ± 0.002	6.8 ± 0.6	1.1 ± 0.08	0.32 ± 0.04	0.21 ± 0.02	14.8 ± 1.1	0.07 ± 0.01	LOD	LOD	6.0 ± 0.5	0.19 ± 0.02
INF-T6	Tap water	70	5		LOD	LOD	5.1 ± 0.5	LOD	LOD	0.15 ± 0.02	LOD	0.04 ± 0.01	LOD	LOD	4.8 ± 0.4	0.11 ± 0.01
INF-T7		80	7		LOD	0.006 ± 0.001	LOD	0.7 ± 0.06	0.22 ± 0.03	LOD	9.6 ± 0.7	0.06 ± 0.01	LOD	LOD	5.6 ± 0.4	LOD
INF-T8		85	10		0.10 ± 0.012	LOD	7.4 ± 0.6	1.3 ± 0.09	0.36 ± 0.04	0.24 ± 0.02	16.5 ± 1.3	LOD	LOD	LOD	7.2 ± 0.6	0.22 ± 0.02
INF-T9		90	12		0.16 ± 0.015	0.011 ± 0.002	9.2 ± 0.8	1.6 ± 0.10	0.48 ± 0.05	0.38 ± 0.03	21.9 ± 1.7	0.11 ± 0.01	LOD	LOD	8.5 ± 0.7	0.34 ± 0.03
INF-T10		95	15		0.23 ± 0.021	0.015 ± 0.003	11.5 ± 1.0	2.0 ± 0.14	0.62 ± 0.06	0.52 ± 0.04	28.4 ± 2.1	0.16 ± 0.02	LOD	LOD	9.8 ± 0.8	0.41 ± 0.03
INF-C1	Ultra-pure	70	5	*T. cordata*	LOD	LOD	LOD	LOD	LOD	LOD	LOD	LOD	LOD	LOD	2.8 ± 0.2	LOD
INF-C2		80	7		LOD	0.006 ± 0.001	4.1 ± 0.4	LOD	0.24 ± 0.03	0.10 ± 0.01	5.5 ± 0.5	LOD	LOD	LOD	LOD	0.07 ± 0.01
INF-C3		85	10		LOD	LOD	5.6 ± 0.5	0.9 ± 0.07	LOD	0.18 ± 0.02	11.8 ± 0.9	0.05 ± 0.01	LOD	LOD	4.9 ± 0.4	0.15 ± 0.01
INF-C4		90	12		0.14 ± 0.016	0.009 ± 0.002	8.3 ± 0.7	1.4 ± 0.10	0.38 ± 0.04	0.29 ± 0.03	18.3 ± 1.4	0.09 ± 0.01	LOD	LOD	6.3 ± 0.5	0.23 ± 0.02
INF-C5		95	15		0.18 ± 0.020	0.013 ± 0.002	10.9 ± 1.0	1.9 ± 0.13	0.55 ± 0.05	0.41 ± 0.03	24.6 ± 1.9	0.14 ± 0.02	LOD	LOD	8.0 ± 0.6	0.36 ± 0.03
INF-C6	Tap water	70	5		LOD	LOD	LOD	LOD	0.22 ± 0.03	0.22 ± 0.02	LOD	LOD	LOD	LOD	5.1 ± 0.4	0.10 ± 0.01
INF-C7		80	7		LOD	LOD	5.2 ± 0.4	0.8 ± 0.06	LOD	LOD	13.2 ± 1.0	0.07 ± 0.01	LOD	LOD	6.7 ± 0.5	LOD
INF-C8		85	10		0.13 ± 0.015	0.010 ± 0.002	7.9 ± 0.6	1.2 ± 0.09	0.41 ± 0.05	0.34 ± 0.03	20.7 ± 1.6	0.11 ± 0.01	LOD	LOD	8.4 ± 0.7	0.28 ± 0.02
INF-C9		90	12		0.21 ± 0.021	0.014 ± 0.002	9.8 ± 0.9	1.7 ± 0.11	0.60 ± 0.06	0.50 ± 0.04	26.9 ± 2.0	0.18 ± 0.02	LOD	LOD	9.7 ± 0.8	0.39 ± 0.03
INF-C10		95	15		0.31 ± 0.029	LOD	13.4 ± 1.2	2.4 ± 0.15	0.78 ± 0.07	0.67 ± 0.05	33.1 ± 2.4	0.24 ± 0.02	LOD	LOD	11.2 ± 0.9	0.46 ± 0.03
INF-P1	Ultra-[illegible]	70	5	*T.* [illegible]	LOD	LOD	3.8 ± 0.3	LOD	LOD	0.09 ± 0.01	LOD	LOD	LOD	LOD	LOD	0.06 ± 0.01
INF-P2		80	7		LOD	LOD	LOD	0.6 ± 0.05	LOD	0.14 ± 0.02	7.4 ± 0.6	LOD	LOD	LOD	3.9 ± 0.3	0.09 ± 0.01

INF-P3		85	10	LOD	0.006 ± 0.001	6.1 ± 0.5	0.9 ± 0.07	0.28 ± 0.03	LOD	12.9 ± 1.0	0.04 ± 0.01	LOD	LOD	5.0 ± 0.4	LOD
INF-P4		90	12	0.13 ± 0.015	0.008 ± 0.001	7.3 ± 0.6	1.3 ± 0.09	LOD	0.26 ± 0.02	19.8 ± 1.5	0.08 ± 0.01	LOD	LOD	6.2 ± 0.5	0.20 ± 0.02
INF-P5		95	15	0.16 ± 0.018	0.011 ± 0.002	9.5 ± 0.8	1.7 ± 0.11	0.42 ± 0.05	0.37 ± 0.03	23.7 ± 1.8	0.13 ± 0.02	LOD	LOD	7.6 ± 0.6	0.32 ± 0.03
INF-P6	Tap water	70	5	LOD	LOD	LOD	0.7 ± 0.06	0.25 ± 0.03	LOD	10.5 ± 0.8	0.05 ± 0.01	LOD	LOD	5.3 ± 0.4	0.12 ± 0.01
INF-P7		80	7	LOD	0.007 ± 0.001	5.8 ± 0.5	LOD	0.34 ± 0.04	0.21 ± 0.02	LOD	0.06 ± 0.01	LOD	LOD	6.5 ± 0.5	0.16 ± 0.01
INF-P8		85	10	0.11 ± 0.013	LOD	8.4 ± 0.7	1.4 ± 0.10	LOD	0.33 ± 0.03	18.6 ± 1.4	0.10 ± 0.01	LOD	LOD	7.9 ± 0.6	0.27 ± 0.02
INF-P9		90	12	0.18 ± 0.017	0.012 ± 0.002	10.7 ± 0.9	1.9 ± 0.12	0.50 ± 0.05	0.46 ± 0.04	25.8 ± 2.0	0.15 ± 0.02	LOD	LOD	9.1 ± 0.7	0.38 ± 0.03
INF-P10		95	15	0.27 ± 0.023	0.016 ± 0.003	12.9 ± 1.1	2.3 ± 0.15	0.70 ± 0.06	0.60 ± 0.05	31.4 ± 2.3	0.20 ± 0.02	LOD	LOD	10.5 ± 0.8	0.44 ± 0.03
Mean				0.17	0.0098	7.71	1.33	0.42	0.30	17.37	0.10	0.000	0.000	6.66	0.226
SD				0.07	0.00334	2.92	0.55	0.17	0.17	8.37	0.06	0.000	0.000	2.22	0.130
RSD%				35.8	34.0	37.8 %	41.6 %	40.7 %	56.3 %	48.2 %	57.4 %	0.000	0.000	33.3	57.4
CV (%)				35.8	34.0	37.8 %	41.6 %	40.7 %	56.3 %	48.2 %	57.4 %	0.000	0.000	33.3	57.4
Min.				0.10	0.006	3.20	0.60	0.22	0.08	4.8	0.03	0.000	0.000	2.8	0.06
Max.				0.31	0.016	13.40	2.40	0.78	0.67	33.1	0.24	0.000	0.000	11.2	0.46
T. tomentosa				0.15	0.0092	6.52	1.08	0.36	0.24	15.21	0.077	0.000	0.000	6.08	0.196
T. cordata				0.21	0.0104	8.27	1.42	0.50	0.34	19.26	0.125	0.000	0.000	7.01	0.255
T. platyphyllos				0.18	0.0100	7.63	1.29	0.41	0.31	18.76	0.101	0.000	0.000	6.89	0.227
Ultra-pure				0.13	0.0084	6.41	1.09	0.33	0.21	10.35	0.07	0.000	0.000	5.29	0.164
Tap water				0.21	0.0114	8.52	1.33	0.48	0.36	22.68	0.123	0.000	0.000	7.75	0.283

Table S7. Transfer Efficiency (%) in *Tilia tomentosa* Infusions Prepared Under Different Brewing Conditions

Sample	Water	Temp	Time	Pb	Cd	Zn	Cu	Ni	Cr	Mn	Co	As	Hg	Al	V
INF-T1	Ultra	70	5	0.00	0.05	0.00	0.59	0.00	0.00	0.52	0.00	0	0	0.00	1.18
INF-T2	Ultra	80	7	0.00	0.00	0.87	0.00	0.00	0.19	0.00	0.09	0	0	0.50	0.00
INF-T3	Ultra	85	10	0.00	0.00	0.00	0.00	0.00	0.27	0.70	0.00	0	0	0.60	1.97
INF-T4	Ultra	90	12	0.00	0.05	1.22	0.79	7.91	0.00	1.10	0.15	0	0	0.73	2.76
INF-T5	Ultra	95	15	18.80	0.08	1.84	1.09	10.12	0.47	1.60	0.21	0	0	0.86	3.75
INF-T6	Tap	70	5	0.00	0.00	1.38	0.00	0.00	0.36	0.00	0.13	0	0	0.69	2.16
INF-T7	Tap	80	7	0.00	0.05	0.00	0.65	6.78	0.00	1.08	0.19	0	0	0.81	0.00
INF-T8	Tap	85	10	15.66	0.00	2.00	1.21	11.09	0.58	1.86	0.00	0	0	1.05	4.32
INF-T9	Tap	90	12	25.09	0.08	2.48	1.49	14.81	0.91	2.47	0.23	0	0	1.24	6.69
INF-T10	Tap	95	15	36.12	0.10	3.11	1.87	19.14	1.25	3.20	0.34	0	0	1.43	8.06

Table S8. Transfer Efficiency (%) in *Tilia cordata* Infusions Prepared Under Different Brewing Conditions

Sample	Water	Temp	Time	Pb	Cd	Zn	Cu	Ni	Cr	Mn	Co	As	Hg	Al	V
INF-C1	Ultra	70	5	0.00	0.00	0.00	0.00	0.00	0.00	0.00	0.00	0	0	1.06	0.00
INF-C2	Ultra	80	7	0.00	0.02	1.52	0.00	13.25	0.24	0.65	0.00	0	0	0.00	0.06
INF-C3	Ultra	85	10	0.00	0.00	2.08	0.96	0.00	0.42	1.40	0.21	0	0	0.74	0.32
INF-C4	Ultra	90	12	20.69	0.03	3.09	1.48	19.75	0.68	2.18	0.39	0	0	0.95	0.49
INF-C5	Ultra	95	15	26.69	0.04	4.04	2.01	28.61	0.97	2.93	0.61	0	0	1.21	0.77
INF-C6	Tap	70	5	0.00	0.00	0.00	0.00	12.50	0.82	0.00	0.00	0	0	0.77	0.84
INF-C7	Tap	80	7	0.00	0.00	1.92	0.86	0.00	0.00	1.71	0.30	0	0	1.01	0.00
INF-C8	Tap	85	10	19.25	0.03	2.91	1.29	28.91	0.98	2.68	0.47	0	0	1.26	2.36
INF-C9	Tap	90	12	31.06	0.04	3.62	1.83	42.30	1.45	3.48	0.78	0	0	1.46	3.29
INF-C10	Tap	95	15	45.80	0.00	4.96	2.59	55.01	1.94	4.29	1.04	0	0	1.68	3.89

Table S9. Transfer Efficiency (%) in *Tilia platyphyllos* Infusions Prepared Under Different Brewing Conditions

Sample	Water	Temp	Time	Pb	Cd	Zn	Cu	Ni	Cr	Mn	Co	As	Hg	Al	V
INF-P1	Ultra	70	5	0.00	0.00	1.52	0.00	0.00	0.24	0.00	0.00	0	0	0.00	0.35
INF-P2	Ultra	80	7	0.00	0.00	0.00	0.70	0.00	0.37	0.85	0.00	0	0	0.59	0.52
INF-P3	Ultra	85	10	0.00	0.01	2.44	0.94	13.25	0.00	1.47	0.11	0	0	0.76	0.00
INF-P4	Ultra	90	12	20.54	0.02	2.93	1.35	0.00	0.83	2.25	0.22	0	0	0.94	1.19
INF-P5	Ultra	95	15	25.12	0.02	3.82	1.76	19.71	1.19	2.69	0.36	0	0	1.16	1.90
INF-P6	Tap	70	5	0.00	0.00	0.00	0.73	11.82	0.00	2.75	0.15	0	0	0.90	0.52
INF-P7	Tap	80	7	0.00	0.02	2.30	0.00	16.09	0.68	0.00	0.19	0	0	1.11	0.69
INF-P8	Tap	85	10	13.90	0.00	3.34	1.91	0.00	1.07	4.88	0.32	0	0	1.35	1.16
INF-P9	Tap	90	12	22.73	0.03	4.25	2.59	10.14	1.49	6.75	0.47	0	0	1.56	1.63
INF-P10	Tap	95	15	34.12	0.03	5.12	3.13	14.22	1.94	8.21	0.63	0	0	1.81	1.98

Table S10. Sampling locations, species identification, and associated biological and soil samples of *Tilia* trees

Tree ID	Species	Latitude (°N)	Longitude (°E)	Sampling date	Time	Area	Site type	Soil sample ID	Bark	Leaves	Flowers
1	*Tilia cordata*	47.04409	21.91902	19.06.2025	10:00	Oradea	Urban	6	Yes	Yes	Yes
2	*Tilia platyphyllos*	46.92496	22.02560	28.06.2025	12:00	Mierlău	Locality	8	Yes	Yes	Yes
3	*Tilia cordata*	46.99047	22.02396	28.06.2025	08:00	Băile 1 Mai	Forest	9	Yes	Yes	Yes
4	*Tilia tomentosa*	46.99601	22.00010	28.06.2025	10:00	Băile 1 Mai	Locality	17	Yes	Yes	Yes
5	*Tilia tomentosa*	47.06824	21.92436	26.06.2025	09:00	Oradea	Urban	29	Yes	Yes	Yes
6	*Tilia platyphyllos*	47.06342	21.97818	26.06.2025	10:00	Oradea	Urban	32	Yes	Yes	Yes
7	*Tilia tomentosa*	46.98907	22.02400	28.06.2025	09:00	Băile 1 Mai	Forest	20	Yes	Yes	Yes
8	*Tilia platyphyllos*	46.92789	21.96047	28.06.2025	13:00	Boboștea	Forest	21	Yes	Yes	Yes
9	*Tilia tomentosa*	46.99886	21.97733	28.06.2025	11:30	Băile Felix	Forest	23	Yes	Yes	Yes
10	*Tilia tomentosa*	46.98960	22.02401	28.06.2025	09:15	Băile 1 Mai	Forest	1	Yes	Yes	Yes
11	*Tilia tomentosa*	46.92474	22.02544	28.06.2025	12:00	Mierlău	Locality	19	Yes	Yes	Yes
12	*Tilia tomentosa*	47.07000	22.01672	26.06.2025	14:00	Oradea–Podgoria	Forest	26	Yes	Yes	Yes
13	*Tilia tomentosa*	47.05524	21.91768	26.06.2025	07:30	Oradea	Urban	30	Yes	Yes	Yes

14	*Tilia platyphyllos*	47.05834	21.91790	25.06.2025	12:00	Oradea	Urban	31	Yes	Yes	Yes
15	*Tilia tomentosa*	47.07063	21.91079	26.06.2025	12:00	Oradea	Urban	7	Yes	Yes	Yes
16	*Tilia tomentosa*	47.04845	21.93011	25.06.2025	09:30	Oradea	Urban	25	Yes	Yes	Yes
17	*Tilia cordata*	47.07723	21.91123	19.06.2025	11:00	Oradea	Urban	2	Yes	Yes	Yes
18	*Tilia tomentosa*	47.07683	21.91063	19.06.2025	08:00	Oradea	Urban	4	Yes	Yes	Yes
19	*Tilia cordata*	47.06381	21.95123	21.06.2025	09:30	Oradea	Urban	13	Yes	Yes	Yes
20	*Tilia tomentosa*	47.07706	21.91181	19.06.2025	12:00	Oradea	Urban	5	Yes	Yes	Yes
21	*Tilia platyphyllos*	47.05478	21.94795	23.06.2025	08:00	Oradea	Urban	12	Yes	Yes	Yes
22	*Tilia tomentosa*	47.06562	21.97427	21.06.2025	10:00	Oradea	Urban	16	Yes	Yes	Yes
23	*Tilia tomentosa*	47.06519	21.93730	21.06.2025	08:00	Oradea	Urban	10	Yes	Yes	Yes
24	*Tilia cordata*	47.06575	21.96003	21.06.2025	09:45	Oradea	Urban	11	Yes	Yes	Yes
25	*Tilia cordata*	46.99588	22.00015	28.06.2025	10:30	Băile 1 Mai	Locality	18	Yes	Yes	Yes
26	*Tilia platyphyllos*	47.04055	21.91774	20.06.2025	–	Oradea	Urban	24	Yes	Yes	Yes
27	*Tilia tomentosa*	47.04419	21.91959	19.06.2025	08:30	Oradea	Urban	3	Yes	Yes	Yes
28	*Tilia cordata*	47.03960	21.92028	19.06.2025	13:00	Oradea	Urban	33	Yes	Yes	Yes
29	*Tilia tomentosa*	47.04102	21.91655	19.06.2025	07:30	Oradea	Urban	27	Yes	Yes	Yes
30	*Tilia tomentosa*	47.06784	21.93270	21.06.2025	07:45	Oradea	Urban	28	Yes	Yes	Yes
31	*Tilia tomentosa*	47.05524	21.94843	22.06.2025	12:00	Oradea	Urban	15	Yes	Yes	Yes
32	*Tilia tomentosa*	47.05613	21.94450	22.06.2025	11:00	Oradea	Urban	15	Yes	Yes	Yes
33	*Tilia tomentosa*	46.99471	21.97821	28.06.2025	12:00	Băile Felix	Locality	22	Yes	Yes	Yes

Note: GPS coordinates are expressed in decimal degrees (WGS84). Each Tree ID (1–34) corresponds to one individual *Tilia* tree from which four sample types were collected: soil, bark, leaves, and flowers. A total of 136 samples were obtained (34 soil, 34 bark, 34 leaf, and 34 flower samples). Soil sample IDs refer to composite rhizosphere soil samples (0–10 cm depth) collected beneath each tree and may differ from Tree IDs due to independent soil sample labeling. Bark, leaf, and flower samples were collected from the same tree and location as the corresponding soil sample. Site type classification includes urban (city), locality (peri-urban), and forest environments. Sampling dates and times refer to field collection, conducted under dry weather conditions. A dash (–) indicates missing or unrecorded data.

Table S11. Supplementary details of soil physicochemical analyses

Parameter	Method / Standard	Operational details
Soil pH	ISO 10390	Measured in distilled water and 1 M KCl at a soil-to-solution ratio of 1:2.5 (w/v); suspension equilibrated for 30 min with intermittent stirring; measurements performed at 20 ± 1 °C
Electrical conductivity (EC)	ISO 11265	Determined in a 1:5 (w/v) soil–water extract after 1 h extraction with deionized water at room temperature
Soil texture	ISO 11277	Pipette–hydrometer method after dispersion with sodium hexametaphosphate; particle-size distribution used for textural class assignment
Soil organic carbon (C_org)	ISO 14235	Walkley–Black dichromate oxidation; suitable for non-carbonate or weakly calcareous soils
Total nitrogen (N_total)	ISO 11261	Kjeldahl digestion–distillation using catalyst tablets; quantification by titration
C/N ratio	Calculated	Calculated from measured C_org and N_total values
Quality control	—	Duplicate analyses, procedural blanks, routine instrument calibration; analytical precision within ±5%

Table S12. Optimized microwave-assisted acid digestion protocols for complete mineralization of solid matrices prior to ICP-MS determination

Matrix	Sample mass (g, d.w.)	Acid composition (Suprapur®)	Digestion step	Temperature ramp (°C·min^{-1})	Target temperature (°C)	Hold time (min)	Applied microwave power (W)	Maximum pressure (bar)	Purpose of step
Soil (mineral-dominated matrix)	0.25–0.50	8 mL HNO_3 (65%) + 2 mL H_2O_2 (30%)	Step 1	Linear (≈12)	120	5	up to 1200	≤40	Initial oxidation and release of exchangeable metals
			Step 2	Linear (≈6)	180	20	up to 1500	≤40	Complete dissolution of silicate-bound and residual fractions
			Cooling	–	<50	20	–	–	Vessel depressurization and stabilization
Bark (lignocellulosic matrix)	0.25	6 mL HNO_3 (65%) + 2 mL H_2O_2 (30%)	Step 1	Linear (≈10)	140	10	up to 1200	≤35	Oxidative breakdown of lignin and cellulose
			Step 2	Linear (≈5)	190	20	up to 1500	≤35	Complete mineralization and metal solubilization
			Cooling	–	<50	20	–	–	Prevention of volatile losses
Leaves (soft plant tissue)	0.25	5 mL HNO_3 (65%) + 2 mL H_2O_2 (30%)	Step 1	Linear (≈15)	130	10	up to 1000	≤30	Rapid oxidation of organic matter

			Step 2	Linear (≈6)	180	15	up to 1200	≤30	Stabilization of digest and matrix clarification
			Cooling	–	<50	15	–	–	Controlled pressure release
Flowers (highly organic, volatile-rich matrix)	0.25	5 mL HNO_3 (65%) + 2 mL H_2O_2 (30%)	Step 1	Linear (≈15)	120	10	up to 1000	≤30	Gentle oxidation to avoid foaming
			Step 2	Linear (≈6)	170	15	up to 1200	≤30	Complete mineralization of organic compounds
			Cooling	–	<50	15	–	–	Preservation of volatile elements (Hg, As)

Table S13. The instrumental settings (a) and data acquisition parameters (b) of the ICP-MS system, which define the operating conditions and analytical procedures for precise metal quantification

**(a) Instrumental parameters**		**(b) Data acquisition parameters for quantitative mode**	
RF power/W	1.4 kW	Measuring mode	Standard (Ar 5.0)<br>Q Cell (Collision Cell) (He 6.0)
Argon (Ar) gas flow, Helium (He) gas flow		Point per peak	3
Nebulizer	1.0 L/min.	Scans/Replicate	7
Plasma gas low rate (Ar 5.0)	18.0 L/min.	Replicate/Sample	7
Auxiliary gas flow rate (He 6.0)	0.20 L/min.		
Lens voltage	37 V	Dwell time (ms)	3
Mirror lens right	32 V		
Mirror lens bottom	31 V		
Sample uptake rate	90 s	Integration time	1-5 ms
Temperature spray chamber		2.10 °C	
Background correction		2 points/peak	
Injector tube		quartz 2-mm id	
Sample cone		Sample Cone 4450	
Skimmer cone		Ni – Skimmer iCAP Q 0.5 mm insert version	
Nebulizer		MicroMist Nebulizer 0.4 mL/min.	

Table S14. Calibration linearity and analytical sensitivity parameters (LoD, LoQ, BEC) for the determination of elements by ICP–MS.

**Element**	**Correlation coefficient**	**LoD (μg/L)**	**LoQ (μg/L)**	**BEC (μg/L)**
^{64}Cu	0.9997	0.035	0.139	0.236
^{65}Zn	0.9999	0.079	1.203	1.310
^{208}Pb	0.9996	0.151	0.231	0.649
^{111}Cd	0.9997	0.007	0.069	0.0031
^{60}Ni	0.9997	0.045	0.181	0.096
^{59}Co	0.9997	0.051	0.136	0.152
^{75}As	0.9999	0.006	0.743	0.018
^{52}Cr	0.9999	1.607	5.533	0.637
^{201}Hg	0.9999	0.043	0.137	0.128
^{55}Mn	0.9998	0.0078	0.0259	0.0418
^{27}Al	0.9996	0.0171	0.0570	0.0287
^{51}V	0.9998	0.0025	0.0083	0.0054

LoD = Detection limit; LoQ = Quantification limit; BEC = Background equivalent concentration.

Table S15. Experimental conditions for the preparation of *Tilia* flower infusions

Parameter	Experimental specification
Tilia species	*Tilia tomentosa, Tilia cordata, Tilia platyphyllos*
Plant material	Dried flowers, finely ground
Sample mass	1.00 g ± 0.01 g
Water volume	250 mL

Water type	Ultrapure water (Milli-Q®); tap water
Infusion temperature range	70–95 °C
Investigated temperatures	70, 80, 85, 90, 95 °C
Infusion time range	5–15 min
Investigated infusion times	5, 7, 10, 12, 15 min
Infusion procedure	Hot water poured over plant material
Post-infusion cooling	To room temperature
Solid–liquid separation	Filtration
Storage container	Pre-cleaned polypropylene vessels
Storage conditions	4 °C until analysis
Replicates	Triplicate for each condition
Quality control	Procedural blanks (water only)

Table S16. Validation parameters of the analytical procedure for the determination of heavy metals (soil) (mg/kg)

Element	**Certified reference material analysis**		**Validation parameters**	
	The result declared by de manufacture	The results obtained in our research	Recovery (%)	Uncertainty (%)
^{208}Pb	18.9 ± 0.5	18.4 ± 0.9	97.4	12
^{111}Cd	0.38 ± 0.01	0.37 ± 0.02	97.4	15
^{65}Zn	106 ± 3	103.2 ± 4.8	97.4	14
^{64}Cu	34.6 ± 0.7	33.8 ± 1.9	97.7	13
^{60}Ni	88 ± 5	86.1 ± 4.1	97.8	16
^{52}Cr	103 ± 4	101.0 ± 3.6	98.1	15
^{55}Mn	538 ± 12	526 ± 21	97.8	14
^{59}Co	13.4 ± 0.7	13.1 ± 0.8	97.8	18
^{75}As	17.7 ± 0.8	17.1 ± 1.1	96.6	17
^{201}Hg	1.40 ± 0.08	1.32 ± 0.09	94.3	13
^{27}Al	7420 ± 210	7210 ± 290	97.2	17
^{51}V	112 ± 5	109 ± 6	97.3	15

SRM – 2709a Standard Reference Material „San Joaquin Soil" was used as certified reference material for method validation.

Table S17. Validation parameters of the analytical procedure for the determination of heavy metals (Tea Leaves) (mg/kg)

Element	**Certified reference material analysis**		**Validation parameters**	
	The result declared by de manufacture	The results obtained in our research	Recovery (%)	Uncertainty (%)
^{208}Pb	0.040 ± 0.003	0.039 ± 0.004	97.5	14
^{111}Cd	0.030 ± 0.002	0.029 ± 0.003	96.7	15
^{65}Zn	30.5 ± 1.2	29.8 ± 1.6	97.7	12
^{64}Cu	6.25 ± 0.30	6.10 ± 0.42	97.6	13
^{60}Ni	2.15 ± 0.15	2.10 ± 0.18	97.7	16
^{52}Cr	1.03 ± 0.08	1.01 ± 0.09	98.1	15
^{55}Mn	720 ± 25	703 ± 31	97.6	14
^{59}Co	0.42 ± 0.03	0.41 ± 0.04	97.6	18
^{75}As	0.28 ± 0.03	0.27 ± 0.03	96.4	17
^{201}Hg	0.017 ± 0.002	0.016 ± 0.002	94.1	13

^{27}Al	520 ± 30	505 ± 38	97.1	16
^{51}V	0.85 ± 0.06	0.83 ± 0.07	97.6	15

NMIJ CRM 7505-a (Tea Leaves) was used as certified reference material for method validation.

Table S18. Validation parameters of the analytical procedure for the determination of heavy metals (in infusions) (μg/L)

Element	Certified reference material analysis		Validation parameters	
	The result declared by de manufacture	The results obtained in our research	Recovery (%)	Uncertainty (%)
^{208}Pb	19.63 ± 0.21	19.1 ± 0.9	97.3	11
^{111}Cd	6.47 ± 0.07	6.30 ± 0.42	97.4	13
^{65}Zn	78.5 ± 1.5	76.6 ± 4.3	97.6	12
^{64}Cu	22.3 ± 0.4	21.8 ± 1.6	97.8	12
^{60}Ni	60.0 ± 1.0	58.6 ± 3.9	97.7	13
^{52}Cr	18.5 ± 0.3	18.1 ± 1.2	97.8	12
^{55}Mn	39.7 ± 0.8	38.7 ± 2.6	97.5	11
^{59}Co	27.1 ± 0.5	26.4 ± 1.9	97.4	13
^{75}As	60.4 ± 0.7	58.8 ± 4.1	97.4	14
^{201}Hg	1.56 ± 0.04	1.47 ± 0.12	94.2	15
^{27}Al	141 ± 3	137 ± 9	97.2	12
^{51}V	37.9 ± 0.6	36.9 ± 2.7	97.4	13

NIST SRM 1643f (Trace Elements in Water) was used as certified reference material for method validation.
